# New insights into the recent collapse of Eastern Baltic cod from historical data on stock health

**DOI:** 10.1371/journal.pone.0286247

**Published:** 2023-05-25

**Authors:** Margit Eero, Keith Brander, Tatjana Baranova, Uwe Krumme, Krzysztof Radtke, Jane W. Behrens

**Affiliations:** 1 National Institute for Aquatic Resources, Technical University of Denmark, Kgs. Lyngby, Denmark; 2 Institute of Food Safety, Animal Health and Environment “BIOR”, Riga, Latvia; 3 Thünen Institute of Baltic Sea Fisheries, Rostock, Germany; 4 National Marine Fisheries Research Institute, Gdynia, Poland; University of Siena, ITALY

## Abstract

The Eastern Baltic cod (*Gadus morhua*) stock is currently in a very poor state, with low biomass and adverse trends in several life history and demographic parameters. This raises concern over whether and to what level recovery is possible. Here, we look for new insights from a historical perspective, extending the time series of various stock health indicators back to the 1940s, i.e. to the beginning of intensive exploitation of the Eastern Baltic cod. The historical data confirm that the stock deterioration in recent years is unprecedented, as all indicators are presently in their worst states on record. Cod body condition and energy reserves were equally low in the 1940s–1950s, accompanied by high parasitic liver worm infection, comparable to that measured in recent years. However, other stock parameters (size structure, size at maturity, stock distribution) are currently in their worst states over the past 80 years. In contrast, the state of cod in the 1970s to early 1990s that is often perceived as a desirable target, was exceptional, with the most favorable indicator levels in the time series. Long-term observation data reveal concurrent or asynchronous trends in different indicators of stock health and to what extent these have coincided with changes in possible external drivers. In this way, the extended time series contribute to ongoing research on understanding the collapse of the cod and its recovery potential.

## Introduction

For centuries, major declines in exploited fish resources have affected human communities that depended on them, caused concerns about the state of marine ecosystems, and stimulated debate and action to bring about fish stock recovery [1–4]. Excessive catches have in many cases been identified as the cause of collapse, but reducing or even banning fishing has not always resulted in recovery, for reasons, which are often not well understood [5–7]. The processes involved could relate to deteriorating climate conditions and/or changes in other natural and anthropogenic drivers altering marine ecosystems [8–10]. Changes in ecosystem conditions that affect fish productivity also challenge the choice of appropriate baselines and recovery targets [11], used in managing the resources concerned.

The Atlantic cod (*Gadus morhua*) stock in the Eastern Baltic Sea (EBC) has undergone one of the most notable collapses of a long-lived key predator fish species in recent times. The stock biomass has declined to the lowest level on record [12], accompanied by pronounced deterioration in various biological parameters over the last decade. On average, cod are small, suffer from low body condition and a high load of parasitic nematodes in the liver; they mature at a small size and the distribution range of the stock has contracted to a relatively small area [13–16]. Growth of the fish has declined [17], natural mortality in the stock has markedly increased [12] and there is no surplus production to support fisheries [18]. To protect the stock and aid its recovery, targeted cod fisheries have been banned since 2019. The reasons for the adverse trends in EBC are debated and proposed drivers include poor oxygen conditions, reduced food availability and quality, thiamine deficiency, increasing number of grey seals as well as overall ecosystem change [19–23].

**Fig 1:**
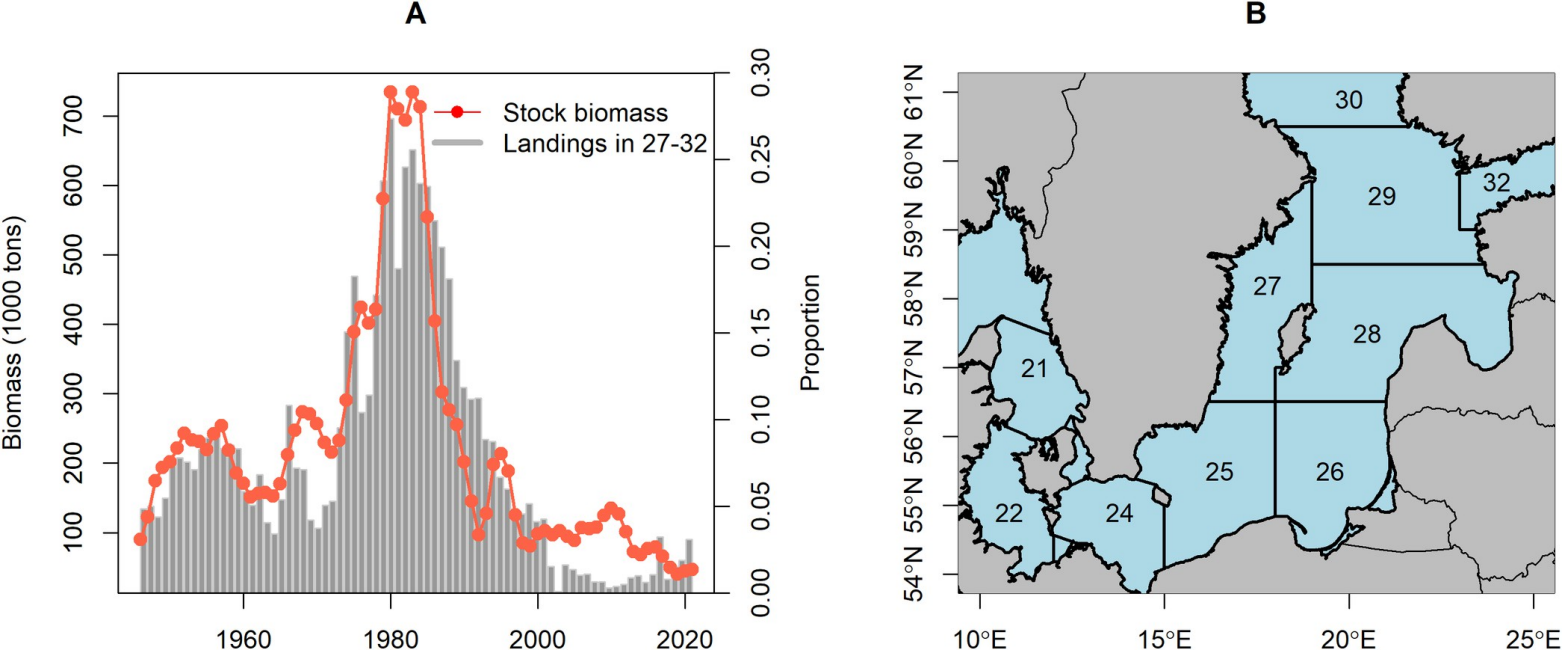
Time series of spatial distribution of Eastern Baltic cod in comparison with stock biomass. (A) Proportion of the commercial cod landings taken in ICES Subdivisions 27–32 (bars), i.e. in the northernmost edge of distribution range of the stock in the Baltic Sea, is considered as a proxy for stock distribution. The biomass shown is for commercial sized cod, i.e. > = 35 cm in length [24] (B) A map illustrating the location of ICES Subdivisions in the Baltic Sea.

Time series of biomass and exploitation intensity of EBC, estimated from stock assessment, are among the longest available for a fish stock in NE Atlantic, extending back to 1946 [12]. Multi-decadal scale information is available also for some other biological parameters of the stock, such as body condition starting from the 1970s [15] and estimates of growth from tagging that have recently been reconstructed back to the 1950s [17]. However, a more comprehensive overview, bringing together information on multiple health aspects of the stock (including size at maturity, size structure etc), is only available since the early 1990s, when standardized international monitoring began. Thus, the much healthier states observed in the early 1990s have come to be regarded as the historic reference for a number of biological parameters, presented in fisheries management advice [24]. The highest level of EBC biomass occurred in the early 1980s (Fig 1), before the major restructuring of the Baltic Sea ecosystem that took place at the end of the 1980s [25, 26]. It is questionable whether recovery to that historic high biomass is possible, and realistic targets in the current state of the ecosystem need to take account of changes in biological parameters affecting stock productivity [27, 28]. These biological parameters, which are indicators of population health, need to be considered alongside the biomass and fishing pressure for policies aiming to promote ecosystem based management, such as the Marine Strategy Framework Directive in Europe [29].

In this paper, we assemble historical information to extend the time series of a range of life history and demographic parameters of the EBC, which represent aspects of population health [28, 30, 31]. Historical perspectives have in many cases provided valuable insights regarding baselines and targets for management and conservation of marine life [32–34]. Our reconstructed data series make available eight decades of observations on a wide range of EBC biological parameters, which is exceptional even compared with the other well-studied North Atlantic cod stocks [35], especially in combination with many other long-term datasets that exist for the Baltic Sea ecosystem and associated pressures [36, 37]. Hence, the longer time series of EBC stock health indicators are considered valuable in adding new perspectives to the perception of the present state of the stock and shed light on realistic recovery expectations. Furthermore, the information made available in this paper contributes a stepping stone for ongoing research and debate to understand the collapse of EBC and define mitigation measures to aid its recovery, as well as providing useful knowledge in relation to similar populations in distress.

## Materials and methods

### Indicators of EBC stock health and data sources

Biomasses, recruitment and fishing mortality estimates of EBC originate from stock assessment starting from 1946 [12]. Since 1991, Baltic International Trawl Surveys (BITS) (https://www.ices.dk/data/data-portals/Pages/DATRAS.aspx) provide data for additional indicators of stock health, such as length structure, body condition and length at maturity, which in later years have contributed background for fisheries management advice for the EBC stock [24]. In this study, we reconstruct the historical developments in EBC body condition, energy reserves, length at first maturity, infection load of parasitic liver worm, length structure and sex ratio in the population and spatial distribution of the stock, focusing on the time period from the 1990s back to the 1940s, i.e. the beginning of intensive exploitation of this stock [38].

For the indicators associated with length structure and spatial distribution of the stock, as well as liver worm load, historical information was derived from published literature and various ICES reports and materials provided to the ICES Working Groups assessing the status of the EBC stock back in time. Time series of average body condition of cod, energy reserves, length at maturity and sex ratio were based on individual fish measurement data (length, body and liver weight, sex, maturity stage). The data for the 1940s–1990s were obtained from the archives of the research institutes in Germany (Thünen Institute of Baltic Sea Fisheries), Latvia (Institute of Food Safety, Animal Health and Environment), Poland (National Marine Fisheries Research Institute) and Denmark (Institute for Aquatic Resources, Technical University of Denmark). The data originated both from sampling of commercial cod catches and from research cruises, and covered ICES Subdivisions (SD) 25–28 (see Fig 1B for location of SDs). Data from commercial fisheries included both sampling of landings in harbours and onboard sampling. With onboard sampling it was not always possible to track whether the sample was from the fraction to be landed or unsorted catch. The data from all sources were treated equally. We acknowledge that commercial fishery could potentially have incentives to target and land healthier individuals. This could bias the average estimates of body condition, as a poor state of this indicator is visually detectable and the fish in poor condition may be discarded. For this reason, we specifically investigated the effect of data source on cod body condition (see the sections below). The individual fish data from 1991 onwards were largely from BITS, supplemented with data from some national cruises and commercial catch sampling for the indicators of body condition and energy reserves. This provided improved seasonal data coverage, and greater sample sizes for larger individuals. In this paper, our main focus is on long-term temporal trends, and we generally do not specifically address spatial variability within the study area (SDs 25–28). An exception is body condition, where we explored possible spatial differences, to supplement earlier analyses by Casini *et al*. [15], where SD specific trends in cod condition since the 1970s were considered.

Information on overall data availability and sample sizes for individual years and indicators is provided in S3 Appendix. Note that the starting years for data availability differ slightly for the different indicators, reflected in the time series presented. The sections below provide further specifics on the data and calculation procedures for each of the indicators included in this study.

### Spatial distribution

Earlier analyses of BITS data documented a contraction of the distribution range of adult EBC in the southern Baltic Sea (mainly ICES SDs 25–26; Fig 1B) in the 1990s–2000s, compared to a wider distribution range of the population in the 1980s [39]. This change in stock distribution is also reflected in commercial fisheries data, with a substantial reduction in the proportion of the cod landings taken in the northeastern areas of the Baltic Sea (SDs 27–32) [40]. We therefore used the proportion of the commercial cod landings taken in ICES SDs 27–32 as a proxy for the relative cod distribution in these areas, since survey data with sufficient spatial coverage were not available for the entire time series we were interested in. Spatially disaggregated data on cod landings were extracted from ICES reports, supplemented by national statistics and literature information for the earliest decades in the time series (Table B in S3 Appendix).

### Body condition

Analyses of body condition used data on total length (L) and total body weight (W) of individual cod (Table A in S3 Appendix). The cod included in the analyses were between 20 and 100 cm in length. Le Cren’s condition index was applied to minimize bias related to fish size [41]. As a first step, data from all years were pooled to estimate the parameters *a* and *b* of the length–weight relationship:

$$W=a*L^{b}$$ (1)

Subsequently, for each individual fish *i*, Le Cren’s condition index *K* was calculated as the ratio between its weight (in g) and the predicted weight of the fish at a given length (in cm) from the length-weight relationship:

$$K_{i}=\frac{W_{i}}{a*L_{i}^{b}}$$ (2)

The individual fish used in the analyses were sampled in different quarters and ICES SDs, and the data sources included both commercial catches and research cruises. Contemporary data indicate that on average cod are in poorer condition in autumn compared to spring [12]. Earlier analyses showed similar trends in cod body condition in all SDs since the 1970s [15], however, spatial heterogeneity in condition could be more pronounced further back in time. Furthermore, the commercial fishery may target cod in healthier condition. To investigate possible effects of these factors on our average condition estimates, we first visually inspected the temporal trends in average body condition separately for each data source (commercial vs survey) and ICES SD (S1 Fig). We present the average condition estimates by quarter, to allow for comparisons with the contemporary data used in ICES, which are for a specific quarter [12]. Additionally, we conducted generalized additive model (GAM) analyses [42] of cod body condition with smoothing spline for year and including quarter, ICES SD and data source as categorical variables:

K∼s(Year)+Quarter+ICESSD+Source (3)

The GAM analysis applied Gaussian distribution, as the condition data were generally normally distributed (Fig A in S1 Appendix). Since few data points were skewed to the right in the distribution curve, we also tried inverse Gaussian distribution, however, this did not change the results (not shown).

### Energy reserves

The ratio of liver weight to body weight, i.e. hepatosomatic index (HSI) is considered as a measure of energy reserves in fish. A decline in HSI in EBC from 1996–2011, along with deteriorating body condition, has been documented [43]. Historical data on EBC cod liver weight are relatively scarce. Individual fish measurements were obtained for a few years in the 1950s, with more regular sampling starting from the 1970s (Table A in S3 Appendix). HSI was calculated for female cod at 40–60 cm in length, similar to an earlier analysis for the recent period [43], to allow for comparability between studies. The index represents the ratio between liver weight (*W*_*L*_) and total body weight (*W*):

$$HSI=100\times \frac{W_{L}}{W}$$ (4)

HSI in fish exhibits an annual cycle associated with reproduction [44]. For EBC, this cycle cannot easily be accounted for by incorporating quarter effects on HSI, as the timing of peak spawning has changed over the years [45]. Furthermore, the seasonal data coverage for HSI is relatively limited. Hence, we present all available data by quarter and focus on detecting major long-term patterns, which appear robust to within-year changes in HSI.

### Length at maturity

Length at maturity (L50) was defined as the length at which half of the fish have become mature. The historical individual fish data used for L50 calculations for the years before the 1990s originated from the 1^st^ and 2^nd^ quarters of the year. From 1991 onwards, data from the 1^st^ quarter BITS were used, as in ICES stock assessments [12]. We also applied the same calculation procedures for L50, as in the ICES assessments for this stock. Based on the maturity stage of individual fish, the fish were classified as mature or immature. The historical maturity assignments had used a scale with 6 or 8 maturity stages. For calculating L50, stages 1–2 were classified as immature and stages 3–8 as mature. The maturity classification was then linked to fish length by applying GLM with binomial form, using a logistic link function, separately for each year. L50 was obtained as a prediction from the model at 50% response probability, for a given year. Data for females and males were combined to increase annual sample sizes. The decline in L50 in last decades has been similar for both sexes [45].

### Sex ratio

Females generally dominate among older cod, as observed in several stocks [35, 46]. This could be caused, for example, by higher spawning mortality in males or by a combined effect of earlier maturation in males and higher fishing pressure on mature fish [46]. For EBC, time series of sex ratios in the population were constructed based on sex information of individual cod in samples (Table A in S3 Appendix), combined for all seasons for which data were available. As a first step, length-specific sex ratios were calculated for all years combined, which showed that females generally start to dominate in length classes > 40 cm, and their proportion in the stock increases with length (S2 Fig). Since we were interested in potential multi-decadal changes in the degree of female dominance among larger cod, we only included cod > 40 cm in length in the sex ratio analyses. We constructed time series of sex ratios both for > 40 cm and > 50 cm cod, to explore the change in female dominance with length.

### Parasitic nematodes in cod livers

We focus on *Contraceacum osculatum* in this study because of the marked increase in infection loads of this parasitic nematode in EBC livers since the 2010s, in parallel to an increase in abundance of grey seals (*Halichoerus grypus*), which are the final host for this parasite in the Baltic Sea [13, 47, 48]. Other parasite species (*Anisakis* sp. and *Pseudoterranowa* spp) have occasionally been found in EBC livers. However, their occurrence in cod in this region is rare compared to *C*. *osculatum*, which is by far the dominant parasite species in EBC livers [22, 48]. Recent studies have shown that cod that are heavily infected with *C*. *osculatum* in their liver have poorer body condition, destroyed liver tissue and show signs of a chronic liver disease, potentially contributing to increased natural mortality [22, 49]. Monitoring of liver worms in cod has hitherto not been part of standard surveys in the Baltic Sea and information is only available from site-specific scientific investigations reported in literature (Table C in S3 Appendix). This allowed for comparison of *C*. *osculatum* loads in cod in recent years with the 1940s–1950s and the 1970s–1980s. Only data from fish above 30 cm in length were included in the analysis, because *C*. *osculatum* is nearly absent in smaller cod. We acknowledge that the investigations conducted over time may differ in terms of spatial coverage, sampling design and methodologies involved, and uncertainty measures associated with the historical estimates are generally not available. Nevertheless, several investigations were often available for adjacent years, providing an indication of consistency of the results for a particular period. Furthermore, to gain confidence in the historical patterns of *C*. *osculatum* load, we used data on both prevalence (the percentage of infected fish in the sample) and intensity of infection (mean number of liver worms in infected individuals). The long-term temporal pattern in *C*. *osculatum* loads emerging from the available studies were compared with trends in grey seal abundance in the entire Baltic Sea [50–52].

### Length structure

Data on length structure of EBC commercial catches in the years 1938–1991 were partly derived from literature reports. In addition, we used the national data that countries historically had provided to the ICES stock assessment working groups in paper form and which had been kept by responsible persons (O. Bagge and E. Ojaveer; pers. comm) (Table D in S3 Appendix). The information was mostly country-specific, in some cases further broken down to sub-areas or quarters, resulting in more than one length composition dataset per year. We treated each dataset separately when calculating length-based indicators, and subsequently averaged the indicator values for a given year. From 2000 onwards, we only used the combined annual length structure of the total commercial catch of the EBC (including discards) from ICES [12].

Length at 95^th^ percentile of a length distribution (L95) was used as an indicator characterizing the proportion of large individuals in the stock [53]. Length-based indicators can generally be sensitive to the strength of incoming year-classes, where an increase in abundance of small fish automatically reduces the proportion of larger fish in the stock. As a sensitivity analysis, we calculated L95 including i) the entire length distribution and ii) only fish > 40 cm in length. This was to explore the effect of smaller cod, whose proportions in the data may vary due to recruitment variability as well as gear selectivity. In our commercial data, 100% selection was usually reached for cod at 40 cm in length. As expected, the values for L95 were lower when using the entire length distribution. However, the trends in L95 were robust to using the entire commercial length distribution or only fish > 40 cm (Fig A in S2 Appendix). We chose to present the second option as a final estimate for L95, where the effects of both recruitment variability and gear selectivity were reduced.

We used length structure information from commercial catches for the entire time series of L95, as very little survey-based information on length structure was available back in time. We acknowledge that length structure of commercial catch is influenced by gear selectivity, in addition to changes in population length structure. We minimized the effect of smaller cod on our final L95 estimates by only using cod > 40 cm in length. However, gear selectivity could potentially also have some impact on the length structure regarding larger individuals. Therefore, we compared our final L95 estimates based on commercial catch data with the ones calculated from BITS from 1991 onwards, supplemented with survey information for some years in the 1950s and 1960s (Table D in S3 Appendix). The L95 estimates based on data from commercial catch and research cruises were similar (Fig B in S2 Appendix), providing assurance that major changes observed in L95 over time reflect changes in population length structure.

## Results

### Spatial distribution

Spatial distribution of commercial landings suggests that the bulk of the EBC stock has been concentrated in the southern Baltic Sea (SDs 25–26) throughout the entire time series, going back to the 1940s. The fraction of the landings taken in the northeastern Baltic Sea (SDs 27–32) was highest in the late 1970s–early 1980s, when it reached up to 25% (Fig 1A). During the 1940s to 1970s, these northeastern areas contributed at most 10% of the landings, and less than 5% since the 2000s. Throughout the time series, the proportion of landings taken in the northeastern Baltic Sea has roughly followed the dynamics of biomass of commercial sized cod (> = 35 cm; Fig 1A), indicating expansion of the stock to the north at higher stock sizes.

### Body condition and energy reserves

**Fig 2:**
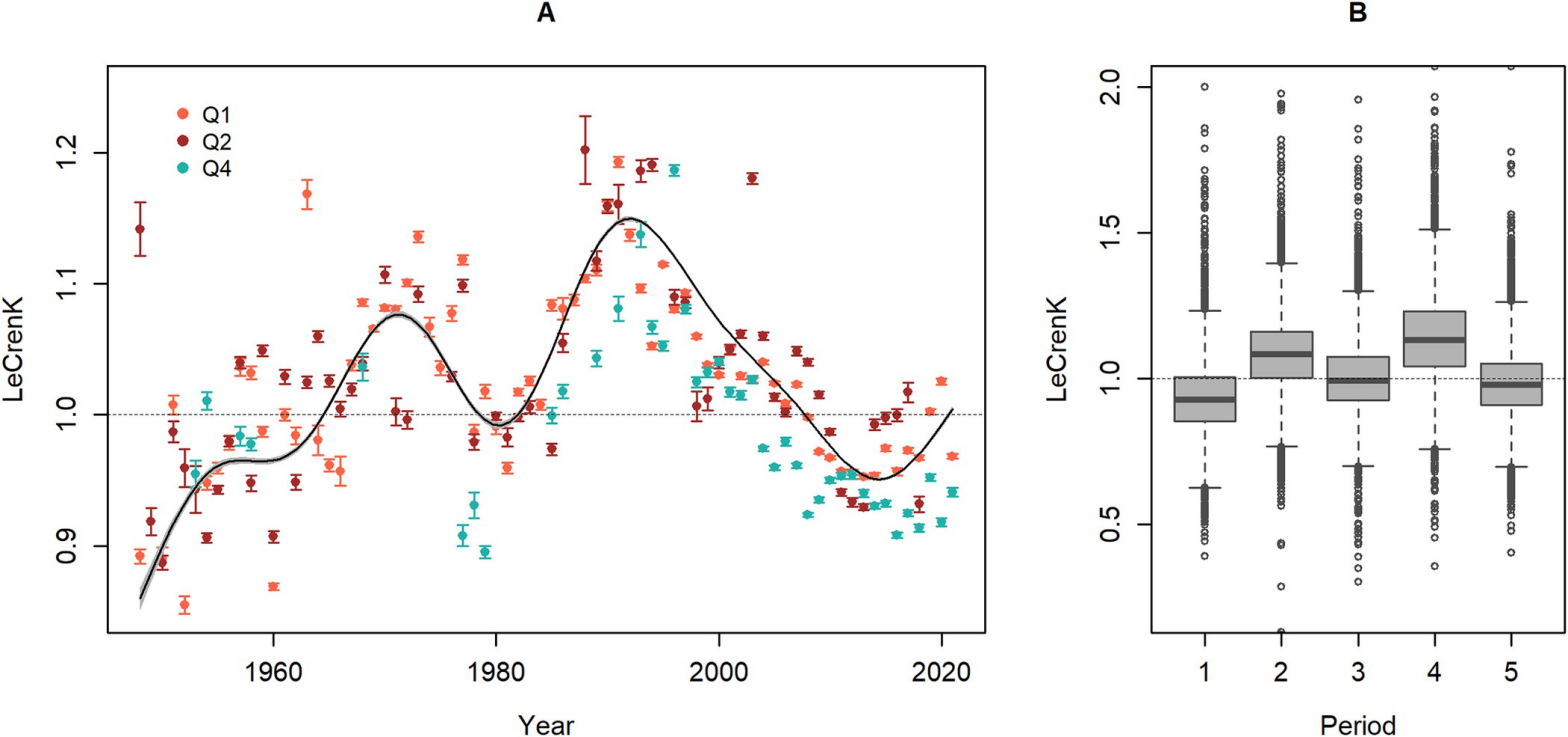
Time series of body condition of Eastern Baltic cod. (A) Developments in average LeCren’s K condition index, by quarter (Q), but combined for all SDs and data sources (commercial and survey). The bars indicate standard error of the mean. The line shows smoothed year effects from GAM analyses including data source, Q and SD as categorical variables. (B) Variability in LeCren’s K condition indices (Q1 and Q2 combined) in selected time periods representing peaks and troughs in the average condition: 1948–1955 (Period 1), 1970–1975 (Period2), 1978–1983 (Period 3); 1989–1993 (Period 4) and 2015–2021 (Period 5).

Long-term trends in the average Le Cren’s condition index *K* of EBC show a gradual increase from low values in the 1940s–1950s to relatively good condition in the early 1970s, followed by a drop in the late 1970s–early 1980s. Thereafter the average body condition quickly improved, reaching a peak in the late 1980s–early 1990s, from which it gradually declined to low level in the 2010s with a slight increase in latest years, especially in Q1 (Fig 2A). GAM analyses showed that data source, SD and quarter (Q) all had significant effects on body condition (S1 Appendix). However, the overall pattern of peaks and troughs in average body condition is apparent in all quarters (Q) (Fig 2A) and SDs for which data are available, and in data from both commercial catches and research surveys (S1 Fig). Notably, the body condition of average cod in Q1–Q2 in 1948–1955 (all SDs and data sources combined) was estimated to be significantly lower (Welch two sample t-test, *p* < 0.001, *t* = 26.803) than the average in recent years (2015–2021). The condition of cod was also relatively low for a short period in the late 1970s–early 1980s, however higher than in recent years (*p* < 0.001, *t* = 13.805). The highest average body condition of EBC in the time series occurred in the late 1980s–early 1990s, significantly higher than during the peak in the early 1970s (*p* < 0.001, *t* = 27.473). Variability in body condition between individual cod has been similarly high throughout the time series (Fig 2B). For example, although cod, on average, were in good condition during the late 1980s–early 1990s, the range of observations included fish in outstandingly good condition as well as those in as poor condition as observed in the 1940s–1950s or in recent years. Likewise, some individuals in good condition were found in, on average, poor condition periods (Fig 2B).

**Fig 3:**
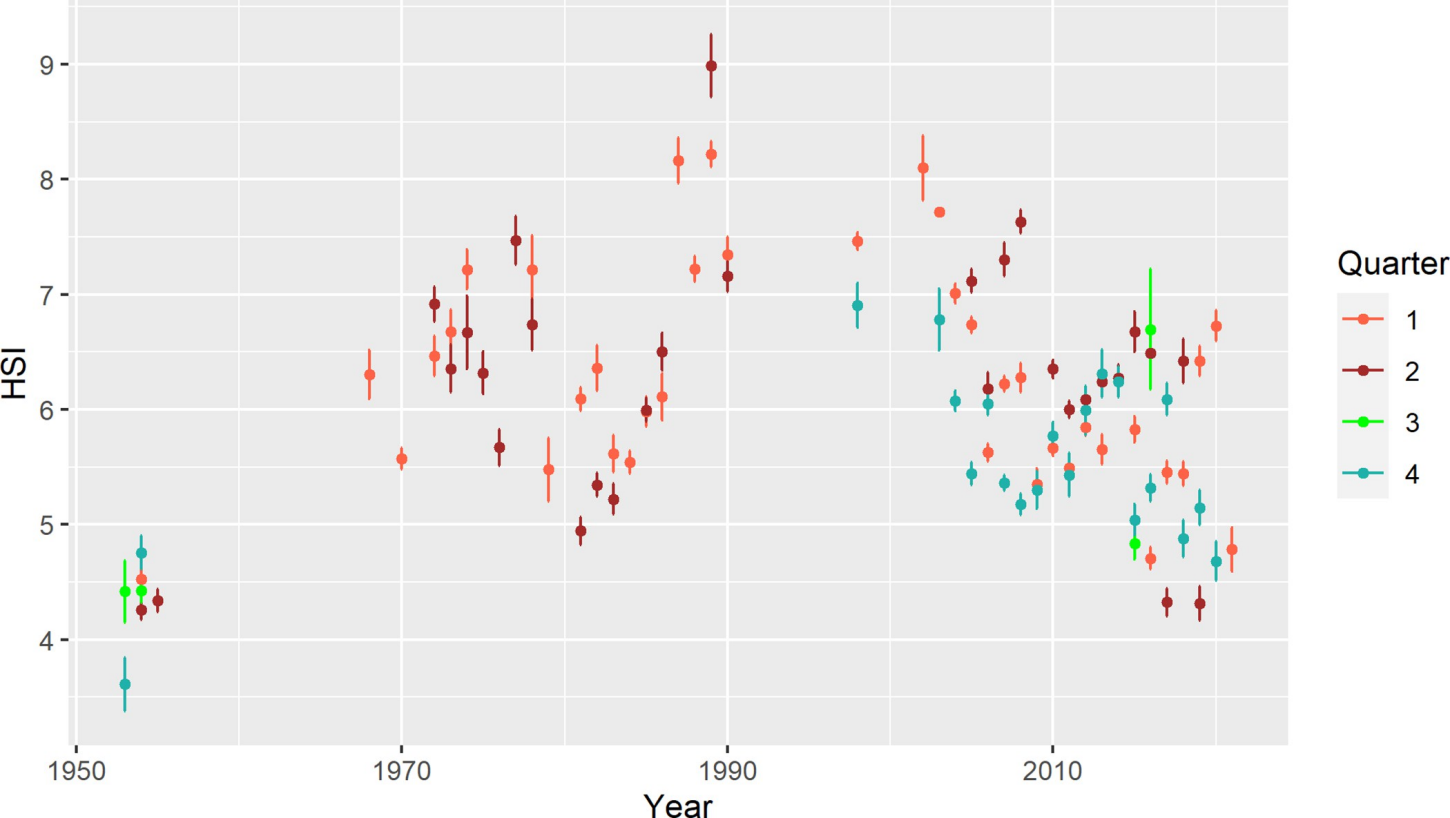
Time series of hepatosomatic index of Eastern Baltic cod. The average index (HSI) is shown for female cod at 40–60 cm in length, by quarter. The bars indicate standard error of the mean.

The available data for hepatosomatic index (HSI) suggest roughly similar long-term patterns in EBC energy reserves as observed for body condition (Fig 3). The average HSI in the first half of the 1950s was at the lowest level on record, consistently for all quarters. Largest sampling sizes in the historical period were available for Q2, where the average HSI in the 1950s (4.29) was comparable with the level of the two lowest HSI values (4.32) observed in contemporary data (in 2017 and 2019). Highest HSI values were apparent in the 1970s and 1990s, interrupted by a drop in the early 1980s, similar to the dynamics of cod body condition. The overall pattern in HSI estimates since the 2000s suggests a declining trend, apart from some relatively high values in some years and quarters.

### Length at maturity and sex ratio

**Fig 4:**
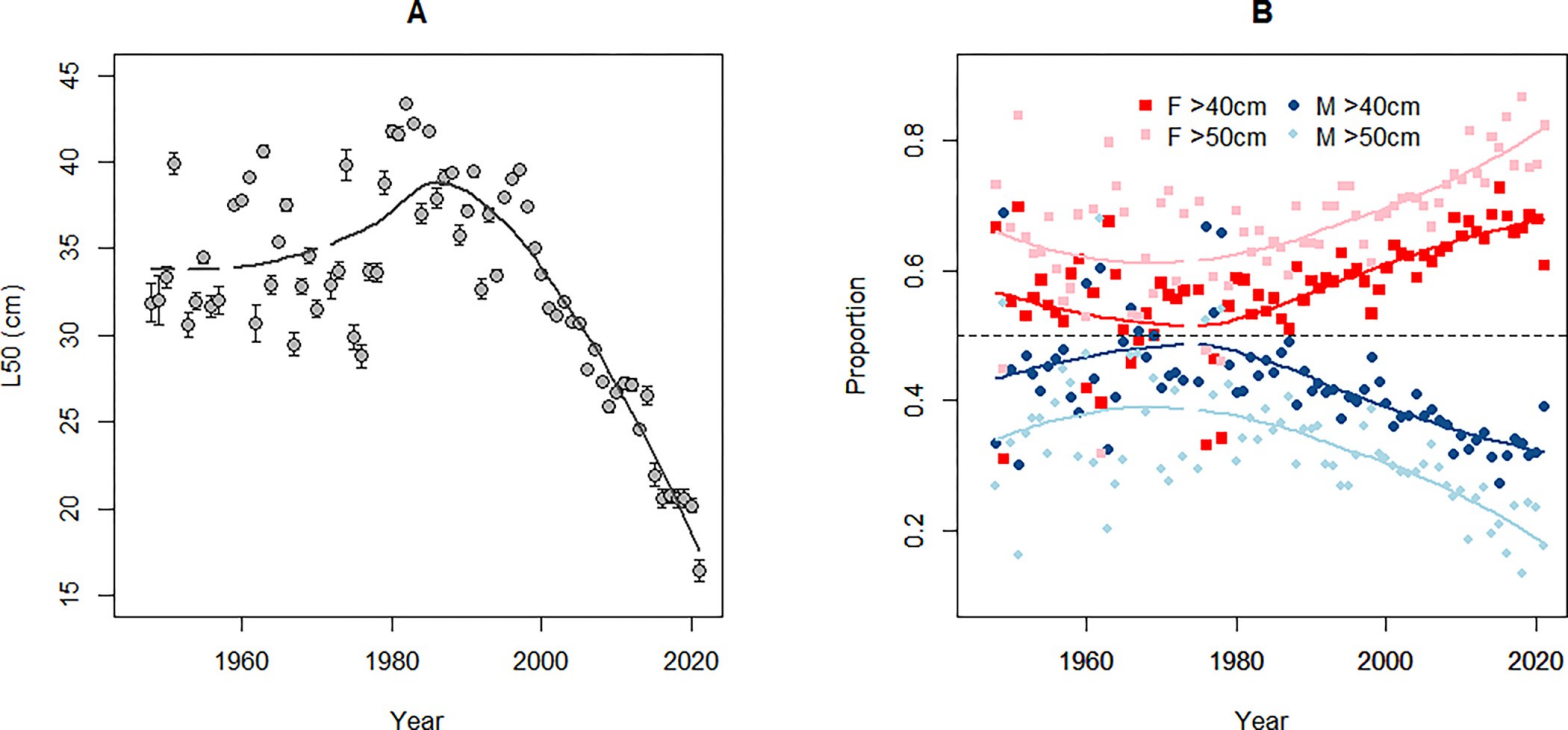
Time series of length at maturity and sex ratio of Eastern Baltic cod. (A) Length at maturity is the length at which 50% of the cod are mature (L50) with the bars indicating standard error of the estimate. (B) Proportion of female (F) and male (M) cod in the stock is shown for fish > 40 cm and > 50 cm in length. The lines in A and B illustrate smoothed trends over time.

L50 of the EBC has declined from around 40 cm in the early 1990s to 20 cm or less in recent years, which is unprecedented in the time series extending back to the late 1940s (Fig 4A). L50 estimates for the 1940s–1980s showed considerable inter-annual variability, but fluctuated mostly in the range of 30–40 cm.

In parallel with the decline in L50 since the 1990s, the proportion of males among larger individuals in the stock (> 40 cm in length) declined substantially (Fig 4B). Females have dominated in this part of the population throughout the time series, however the proportion of males was at least 40% or higher in most years until the 2000s, after which it dropped to around 30% in recent years. A similar or even more pronounced increase in female dominance over time is apparent in cod > 50 cm in length, where the proportion of males has declined from the historical level of 30–40% to around 20% or below in recent years. The proportion of females was also greater in the 1950s, compared to the 1960s–1980s, although as the historical data are relatively noisy, the pattern is not as clear as in recent years.

### Parasitic nematodes in cod livers

**Fig 5:**
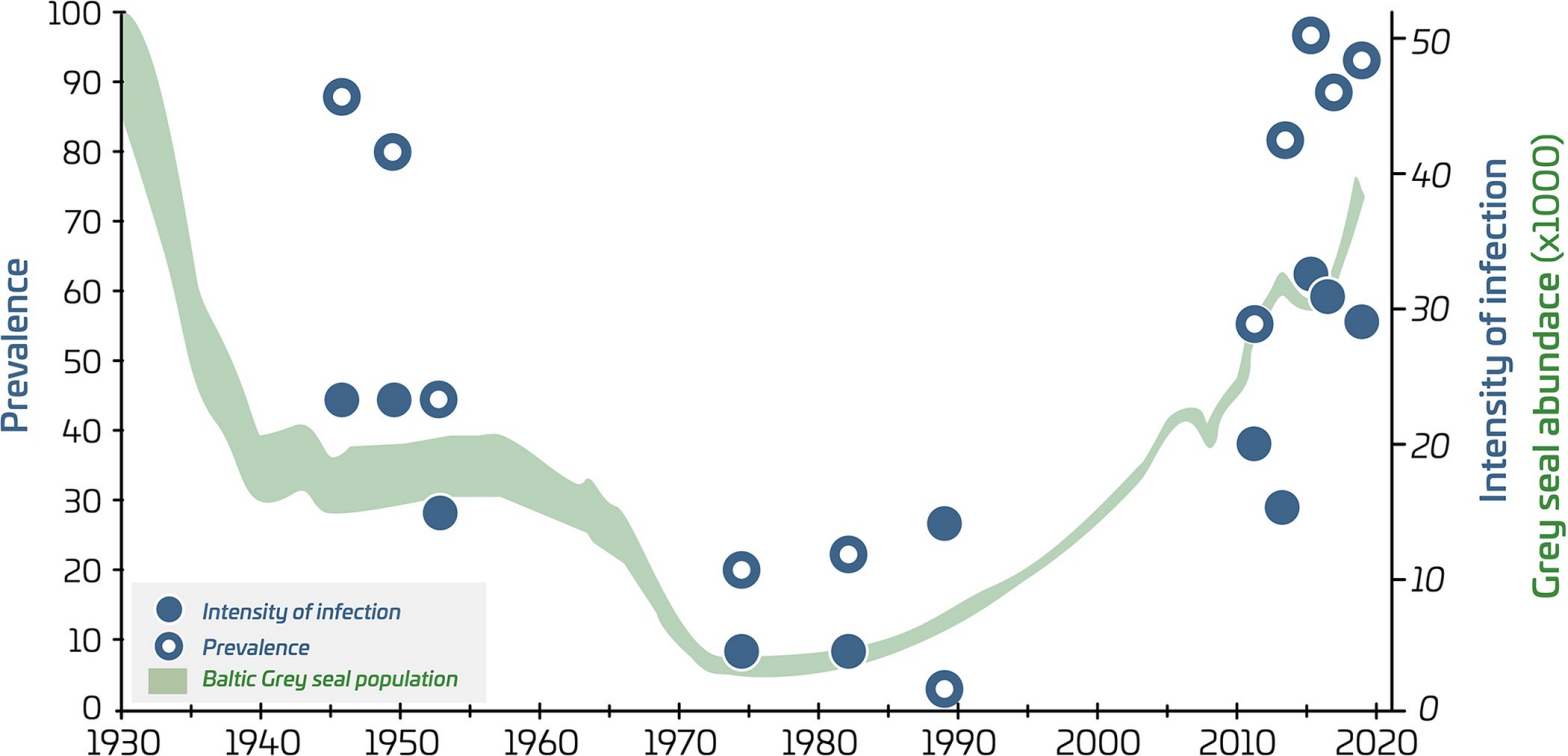
Liver worm (*C*. *osculatum*) infection loads in Eastern Baltic cod in comparison with trends in grey seal abundance in the Baltic Sea. *C*. *osculatum* load is represented by prevalence (percentage of infected fish; open circles) and intensity of infection (mean number of parasites in infected individuals; filled circles). Grey seal abundance shown in the figure is for the entire Baltic Sea, while liver worm infection loads are from the southern and central Baltic Sea.

*C*. *osculatum* loads in EBC were highest in recent years (2016–2020), when most investigated cod were infected (prevalence of 88–100%), with an average infection intensity of 29–33 nematodes per liver (Fig 5). Both prevalence and intensity of infection were somewhat lower in 2012–2015, ranging between 55 and 81% and 16–20 worms per liver, respectively. Studies of *C*. *osculatum* infection from the 1940s estimated that 80–88% of cod were infected, with an average of 23 worms per liver, i.e. similar to recent years. A somewhat lower *C*. *osculatum* load was found in the 1950s, with prevalence of 44% and average infection intensity of 15. Lowest prevalence (2–22%) and intensity (4–14 worms per liver) were recorded in the 1970s and 1980s. Changes in both the prevalence and infection intensity followed roughly similar patterns over time, and coincided with trends in grey seal abundance in the Baltic Sea (Fig 5). Grey seal abundance was high both in recent years (approx. 30.000–40.000 animals) and in the 1940s when the highest *C*. *osculatum* loads were also recorded. In contrast, the grey seal abundance in the Baltic Sea was at a historic low in the 1970s–1980s (approx. 8000 animals), concurrent with the lowest *C*. *osculatum* infection loads reported in cod.

### Length structure

**Fig 6:**
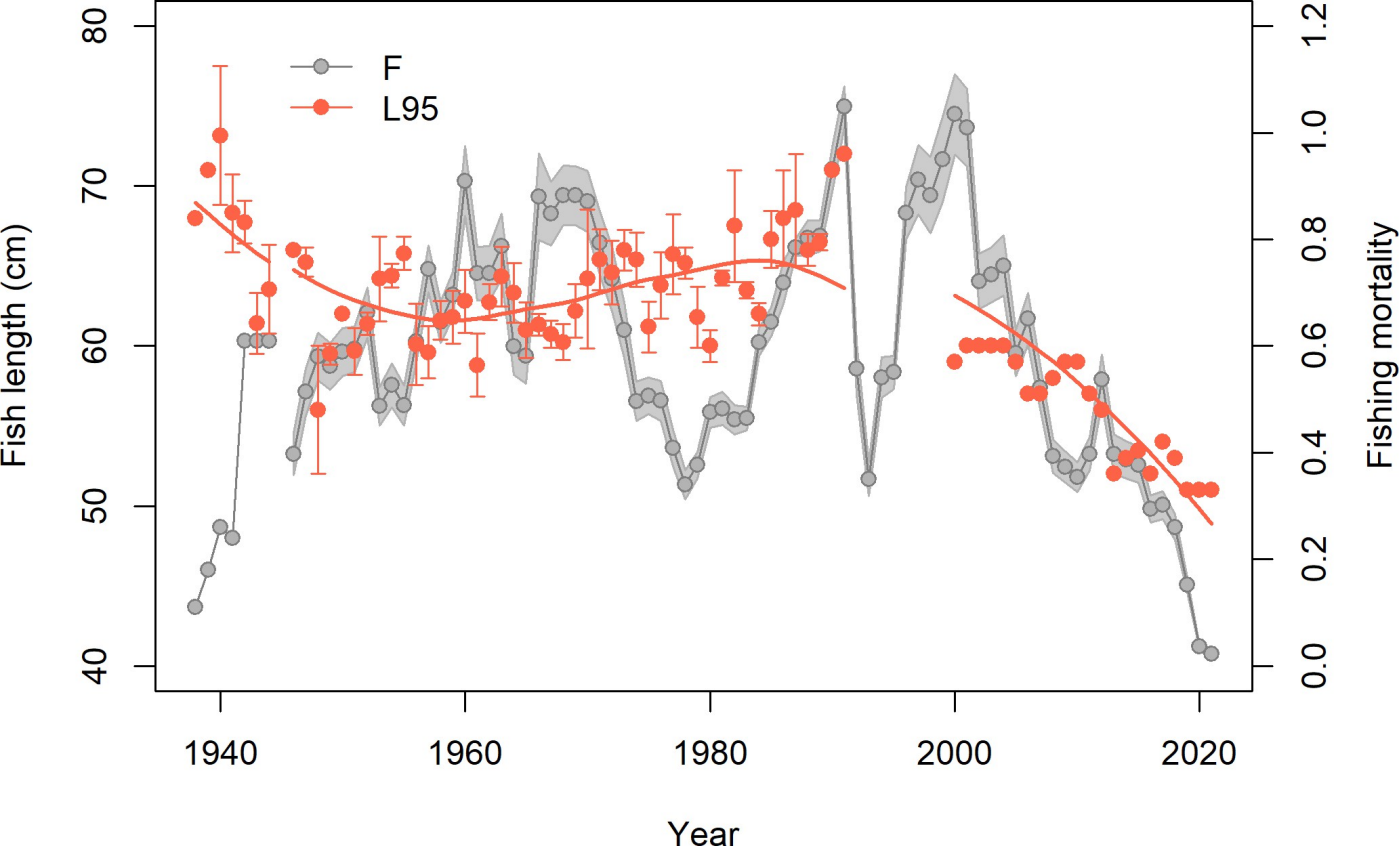
Time series of length structure of Eastern Baltic cod stock in comparison with trends in fishing mortality. L95 is length at 95th percentile of the length distribution, based on data from commercial catches. The error bars show standard error of L95 for years where several length distribution datasets were available. The line illustrates smoothed trends in L95 over time. Fishing mortality (F) is average for ages 4–6, including 90% confidence intervals [12, 38].

The L95 indicator for the EBC stock has declined to around 50 cm in recent years, which is the lowest value in a time series that goes back to the late 1930s (Fig 6). In the late 1930s–early 1940s, L95 was around 70 cm and declined to around 60 cm by the early 1960s. Thereafter, L95 gradually increased, reaching 65 cm or slightly above in the 1980s. Since 2000s, L95 has sharply declined to the present record low level. Historically, before the 2000s, smoothed L95 appears at least to some extent inversely related to fishing mortality (*r*^*2*^ = 0.43, *p* < 0.001). The high values of L95 in the beginning of the time series corresponded to a relatively low fishing mortality. Also, the second peak in L95 in the 1980s occurred simultaneously or slightly after a drop in fishing mortality. In contrast, the decline in L95 since the 2000s occurred in spite of the steep reduction in fishing mortality (Fig 6).

## Discussion

### Historical baselines and perspectives of stock status

The field of marine historical ecology has emerged over the last decades with an aim to provide context for contemporary ocean management [54, 55]. Observations from the past can provide valuable insights in relation to baselines for marine animal populations, and prevent intergenerational loss of information [32–34, 56], notwithstanding that historical data are often less precise and data collection may have followed less rigorous protocols than contemporary monitoring data. The historical data on EBC stock health indicators are no exception, and issues such as relatively low sample sizes and intensity in some years and limited spatial coverage likely introduce some uncertainties in the estimates. Nevertheless, the resulting indicator values were generally consistent between adjacent years. Also, in cases where major long-term trends emerged from the data, these usually showed large enough contrasts between time periods to make it unlikely that the differences are merely due to measurement errors.

**Fig 7:**
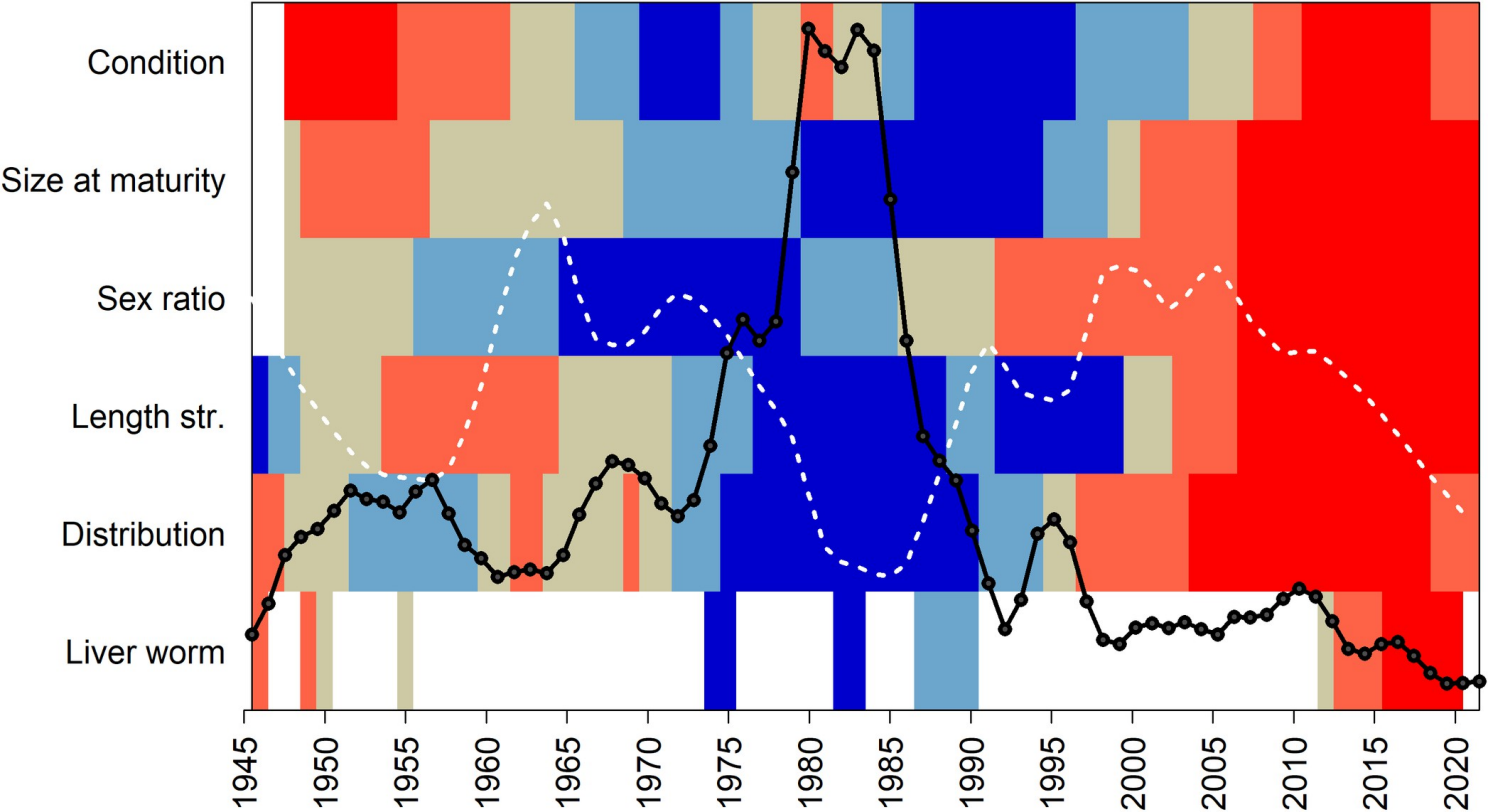
Long-term relative changes in health indicators of Eastern Baltic cod. The colors represent 20^th^ percentiles of the range of smoothed average values observed in the time series, with the blue representing the best and red the worst state. These are overlaid with trends in biomass of commercial sized cod (> = 35 cm; black line) and smoothed time series of recruitment per spawning stock biomass (white broken line), estimated from stock assessment.

Earlier historical data mining efforts enabled reconstruction of the biomass dynamics of EBC back to the onset of intensive exploitation of this stock during the Second WW [38]. The data from 1946 onwards have recently become an integral part of the official stock assessment, used to provide fisheries management advice [24]. The longer time-series of biomass estimates revealed that the record high stock size and associated high catches in the early 1980s, sometimes referred to as a cod-dominated regime [26, 37], were unusual and lasted for a relatively short time. Periods of high stock biomasses are often associated with good recruitment events. This was the case for the EBC in the early 1980s [12] and, for example, for the North Sea gadoid outburst in the early 1970s [57]. In both cases, good recruitment has been ascribed to favorable survival and feeding conditions for early life stages, driven by different environmental and physical processes [58, 59]. The historical data on a wider range of biological parameters indicate that the environmental-ecological conditions that prevailed in the Baltic Sea during the 1970s to early 1990s benefitted the EBC stock more profoundly than just via recruitment. An earlier investigation using tagging data back to the 1950s has demonstrated that growth rates of individual cod were highest in the 1980s [17]. Our analyses show that in this period, the cod stock also displayed a healthy demographic structure, wide distribution range and lower infection loads with *C*. *osculatum*. Furthermore, body condition of cod was good during the 1970s and early 1990s, though interrupted by a period of lower condition in the early 1980s. For some of these indicators, previous studies have provided information extending back from the beginning of BITS in the 1990s. These show, for example, that a peak in body condition in the early 1990s was preceded by lower values in the 1980s and late-1970s [15] and size at maturity was larger in the mid-1980s than in subsequent years [60]. Evaluating the relevance of particular indicator states as baselines or reference conditions, requires time series that are as long as possible. It is also preferable to consider several indicators together rather than basing assessments on single indicators. This provides a more comprehensive evaluation of overall stock status, similar to the use of multiple indicators in assessing the status of ecosystems [61]. From the historical data presented in this paper, it is evident that the concurrence of healthy states of various biological parameters of EBC during the period from the late 1970s to early 1990s was exceptional rather than representing the average historical baseline. It is therefore unrealistic to regard such indicator levels as targets that the stock should recover to and maintain. The indicator levels in recent years provide a sharp contrast, being the most adverse in the time series and confirming that the current poor state of the stock is unprecedented (Fig 7). It is noteworthy that cod body condition, energy reserves and infection load of *C*. *osculatum* were in similar adverse states in the 1940s–1950s, however, the relatively more favorable states of other biological parameters indicate that the stock was overall in a better shape than is presently the case. The period from the 1940s–1970s is generally characterized by an intermediate health status of the stock, and is probably more realistic as a recovery target, as argued by Tomczak *et al*. [37], who suggested this time period as a reference for the Baltic Sea ecosystem.

### Concurrent trends in indicators of stock health and associated external drivers

The dynamics of the EBC stock has long been considered to be mainly determined by fishing, together with direct and indirect effects of salinity and oxygen conditions on recruitment [62, 63]. Consequently, much of the process-oriented research on EBC in the last few decades has focused on the hydrographic drivers of recruitment [58, 64, 65]. The deterioration of cod body condition, length structure and liver worm load has only relatively recently attracted increasing attention to these aspects of population health [13–23, 47–49]. For example, regular monitoring of cod liver worms as part of BITS became mandatory only in 2021. There is so far no consensus on the relative importance of the drivers and mechanisms involved in the poor state of cod, but intense ongoing debate [15, 19, 21, 66, 67]. This resembles the situation of other iconic cod stock collapses, such as the Newfoundland-Labrador stock, where the role of environmental factors, in particular low temperature on prey species (capelin), natural mortality and growth is still contentious and the mechanisms are unclear [68, 69]. Causal links to external drivers and how the different health aspects of a stock interact with one another are generally difficult to disentangle, due to the complexity of the ecological processes involved, requiring experimental approaches. Nevertheless, long time series of observation data can reveal concurrent or asynchronous trends in variables of interest and thereby contribute at least correlation-based evidence that may inspire future dedicated research.

In addition to removing stock biomass, fishing generally truncates population size structure [70, 71]. The long time series of L95 for the EBC provides a rare opportunity to demonstrate how this indicator has both increased and declined over time, roughly inversely to changes in fishing pressure (Fig 6). However, we note that L95 increased in the 1960s–1970s while fishing mortality was high, indicating that fishing was not the only factor affecting L95. Nevertheless, at least some association between L95 and fishing pressure is apparent until the early 2000s (Fig 6), in contrast to other stock indicators that followed a different trajectory, likely influenced by a combination of different drivers. It is notable that since the 2000s, changes in length structure have become decoupled from fishing pressure and have joined the deteriorating path of all other biological parameters, despite a substantial reduction in fishing mortality. A pronounced increase in natural mortality [12] in combination with low growth [17] are likely responsible for the current low proportion of large individuals in the stock. Both the increase in EBC natural mortality and reduction in growth are likely, at least partly, associated with poor body condition and a high *C*. *osculatum* load [22, 49, 72, 73]. Furthermore, the present unprecedentedly small size at maturation (Fig 4A), in combination with poor body condition, is expected to cause mortality due to energy costs associated with reproduction [74, 75]. The increasingly skewed sex ratio in the EBC stock (Fig 4B) in recent years suggests a relatively higher mortality of males among larger cod. We note that the > 40 cm length group used in sex ratio analyses includes a relatively higher proportion of older individuals in recent years than historically, due to reduced growth [17]. As age reading of EBC is problematic [76], it is presently not possible to disentangle to what extent the increasing female dominance in later years reflects changes in age structure or a relatively greater increase in the mortality of males compared to females in the same age. The latter would point at a more semelparous spawning strategy in males, which has been suggested e.g. for capelin when experiencing a high adult mortality [77].

An increase in infections with the trophically transmitted *C*. *osculatum* in EBC livers has been observed in the last decade, concurrent with the increase in the grey seal population in the Baltic Sea [13, 47].

The increased grey seal abundance probably also contributes to natural mortality of cod through predation, however its relative impact is unknown [78]. Several factors likely influence transmission rate of *C*. *osculatum* to cod livers [48], while some association between grey seal abundance and infection rate is to be expected. This is supported by concurrent changes in these variables not only in recent years, but also previously in the historic record. It should be noted that the time-series of grey seal abundance presented in this paper is for the entire Baltic Sea (Fig 5), as these estimates are not available for local areas in the southern and central Baltic Sea, where the studies of cod livers originate from. Grey seal abundance is generally highest in the northern and central Baltic Sea, where their main breeding colonies are located [50, 52]. Thus, an association between total grey seal abundance in the Baltic Sea and liver worm infection load in cod should be treated with some caution. However, grey seals have in last decades recolonized also the southern Baltic Sea, concurrent with the overall population growth [52]. Furthermore, seals are considered highly mobile animals and are likely to visit areas in the southern Baltic Sea, where they are not necessarily breeding [50, 52]. Thus, it is reasonable to assume that the trends in overall abundance of grey seals in the Baltic Sea at least to some extent reflect trends in their occurrence also in areas where transmission of *C*. *osculatum* to cod livers can take place.

Several studies have noted a pattern of high numbers of *C*. *osculatum* in EBC livers coinciding with low body condition of the fish [22, 48, 49, 73]. A connection between these indicators is plausible, as a high average *C*. *osculatum* load in cod corresponded to, on average, low body condition and HSI index both in recent years and in the 1940s–1950s. Furthermore, recent analyses have shown that the lipid content in heavily infected livers was approximately halved compared to the livers of uninfected individuals [22]. A similar pattern was observed in the 1940s [79, 80], potentially underpinning the association between HSI and *C*. *osculatum* load. The degree to which there is a direct causal link between body condition and *C*. *osculatum* load is difficult to prove, as cod that are already in a poor condition may be more susceptible to liver worm–a ‘chicken or the egg’ dilemma. A recent study combining field data and laboratory experiments, however suggests that the metabolic function of the liver is compromised by high parasite loads, which in turn may negatively affect the nutritional condition and overall health of the fish [22].

Although the periods of high average *C*. *osculatum* load in cod have always coincided with periods of low average condition in the available data, the drop in cod condition in the early 1980s occurred nearly in the absence of *C*. *osculatum*. This period corresponded to the peak in cod biomass, so reduced condition in the early 1980s may be attributable to density dependence [15]. Regarding external ecosystem drivers, the biomass of sprat (*Sprattus sprattus*), i.e. the main fish prey for cod, was relatively low in the 1980s [12], and also in the low condition period in the 1940s–1950s [81]. In recent years, availability of sprat for cod is limited due to reduced spatial overlap between the species [15, 43]. Thus, it is plausible that prey availability has been involved in all cases, when deterioration of average condition of EBC has been observed in the time series. Data on benthic prey back in time are scarce, but available proxies suggest relatively low biomasses of at least some species before the 1970s–1980s [37]. The low intake of benthic prey in recent decades [21] is possibly interconnected with hypoxia [19]. Hypoxia, which is among the proposed drivers for the current poor health status of the EBC stock [15, 82] is unlikely to have been responsible for the poor condition of cod in the 1940s–1950s, because the oxygen status in the Baltic Sea was generally considerably better, and only started to gradually deteriorate after the 1950s [83].

These observations suggest that while some drivers and processes may have shaped variations in EBC individual health indicators in a similar way over multiple decades, other impacting factors have likely changed over time. The present exceptionally poor state of the stock on all metrics suggests that more drivers and mechanisms are having simultaneous adverse effects than has been the case in the past. This complicates our understanding of the recovery potential of the stock.

### Future perspectives

The historical data clearly demonstrate that the EBC is currently in an unprecedentedly poor state in terms not only of biomass, but also all other biological parameters that provide an indication of the health of the stock. It is somewhat reassuring that similar poor levels have occurred for some of the indicators (body condition, *C*. *osculatum* load) and subsequently improved, suggesting that recovery is possible. However, the current poor state of all stock parameters may compound the difficulty of recovery of the stock, especially while natural mortality is high [12], which is considered as one of the reasons hampering recovery of other collapsed cod stocks [84].

A downward trend in the number of recruits produced per spawner is a concern for the EBC stock development in coming years (Fig 7). Recruitment variability of EBC is known to be largely environmentally driven [45]. However, with the present poor health status of the fish, parental effects may have become important for reproduction as well, contributing to the generally declining recruitment during the last decade [12]. Parental effects are difficult to document in wild fishes, but research on broodfish in aquaculture has shown that poor nutritional status impairs fecundity, egg quality and embryo development [85]. In the long term, climate change is projected to further deteriorate living conditions for cod in the Baltic Sea [63, 86], reducing its recovery potential.

Complex ecosystem processes seem to have become crucial for the stock development, challenging the management system, which has traditionally focused on fisheries [18]. Dedicated research may eventually improve our understanding of the processes involved and provide better guidance regarding aspects that are at least to some extent under management control. However, to track any progress towards recovery, continued regular monitoring of a wider range of stock health indicators is recommended. It is noteworthy that for EBC, a recovery was declared in the 2000s, in response to an increasing trend in biomass and a decline in fishing mortality estimated from stock assessment [87], which led to substantial increases in fishing quotas during 2009–2014 [24]. Retrospectively seen, the deteriorating states of biological parameters provided warnings that the overall stock status was not improving, but these were overlooked until the recovery signs regarding biomass also vanished. Future stock assessment and management of this and other stocks can learn from such experiences, and consider multiple indicators of stock health, not only biomass, in defining overall stock status and appropriate management measures.

## Supporting information

S1 Fig Body condition of cod shown separately for data sources and ICES Subdivisions. Data sources (SourceID) 1 and 2 refer to commercial and survey data, respectively. On both panels, the dots show mean values of body condition (LeCren’s K) and error bars indicate standard error or the mean.
(TIFF)

S2 Fig Proportions of males and females in Eastern Baltic cod stock, by length. Based on data combined for all years in the time series.
(TIFF)

S1 Appendix Results of GAM analyses of cod body condition. (PDF)

S2 Appendix Sensitivity analyses of L95 indicator. (PDF)

S3 Appendix Data sources and sampling sizes for indicators of Eastern Baltic cod stock health. (PDF)

S4 Appendix Data for indicators of Eastern Baltic cod stock health. (PDF)
