# Supplementary figures and images for: New insights into the recent collapse of Eastern Baltic cod from historical data on stock health
Source: PLoS One. 2023 May 25;18(5):e0286247. doi: 10.1371/journal.pone.0286247 (PMC10212152; doi:10.1371/journal.pone.0286247)

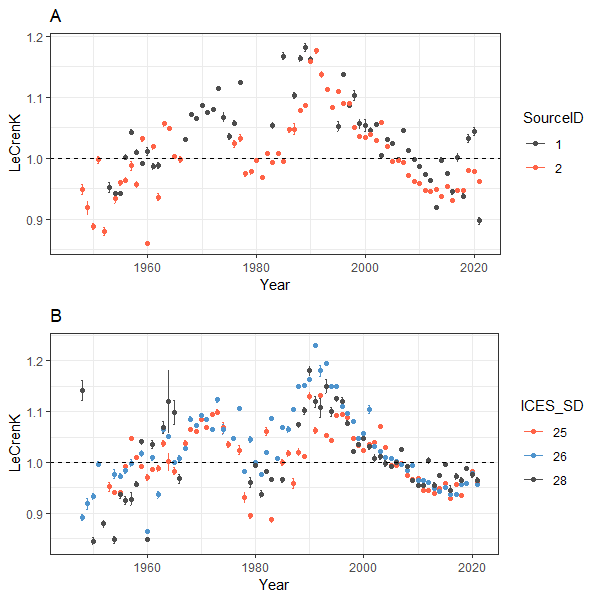

Supplement: S1 Fig — Data sources (SourceID) 1 and 2 refer to commercial and survey data, respectively. On both panels, the dots show mean values of body condition (LeCren’s K) and error bars indicate standard error or the mean. (TIFF) [file pone.0286247.s001.tiff]

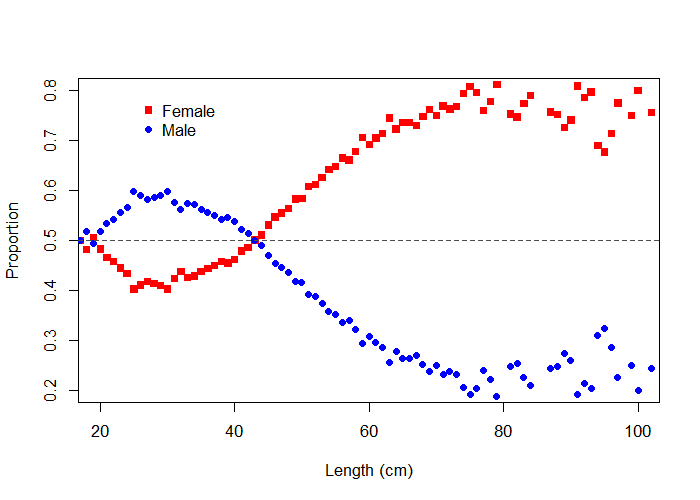

Supplement: S2 Fig — Based on data combined for all years in the time series. (TIFF) [file pone.0286247.s002.tiff]
