# Supplementary material for: New insights into the recent collapse of Eastern Baltic cod from historical data on stock health
Source: PLoS One. 2023 May 25;18(5):e0286247. doi: 10.1371/journal.pone.0286247 (PMC10212152; doi:10.1371/journal.pone.0286247)
Supplement: S1 Appendix — (PDF) [file pone.0286247.s003.pdf]

S1 Appendix. Results from GAM analyses of cod body condition.

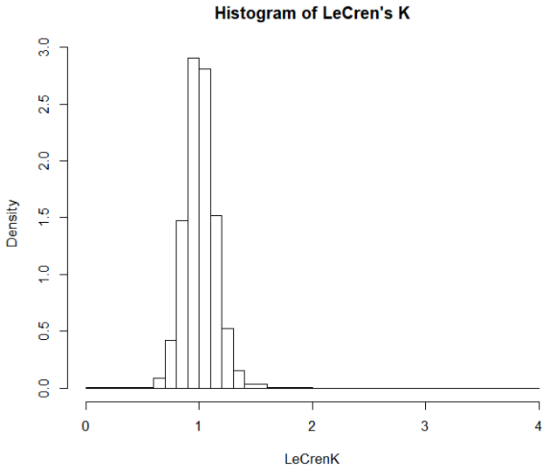

Fig A. Histogram of LeCren's K condition values of cod.

Table A. Statistics from GAM analyses of cod body condition.

Family: gaussian
Link function: identity

Formula:
LeCrenK_TW ~ s(Year) + Quarter + ICES_SD + SourceID

Parametric coefficients:

	Estimate	Std. Error	t value	Pr(>|t|)	
(Intercept)	1.0268318	0.0006169	1664.45	<2e-16	***
Quarter2	0.0105188	0.0006663	15.79	<2e-16	***
Quarter4	-0.0327440	0.0005962	-54.92	<2e-16	***
ICES_SD26	0.0250115	0.0005531	45.22	<2e-16	***
ICES_SD28	0.0150927	0.0009102	16.58	<2e-16	***
SourceID2	-0.0277617	0.0006877	-40.37	<2e-16	***

Signif. codes: 0 '***' 0.001 '**' 0.01 '*' 0.05 '.' 0.1 ' ' 1

Approximate significance of smooth terms:

	edf	Ref.df	F	p-value	
s(Year)	8.999	9	4952	<2e-16	***

Signif. codes: 0 '***' 0.001 '**' 0.01 '*' 0.05 '.' 0.1 ' ' 1

R-sq.(adj) = 0.15 Deviance explained = 15%
GCV = 0.01814 Scale est. = 0.018139 n = 330950

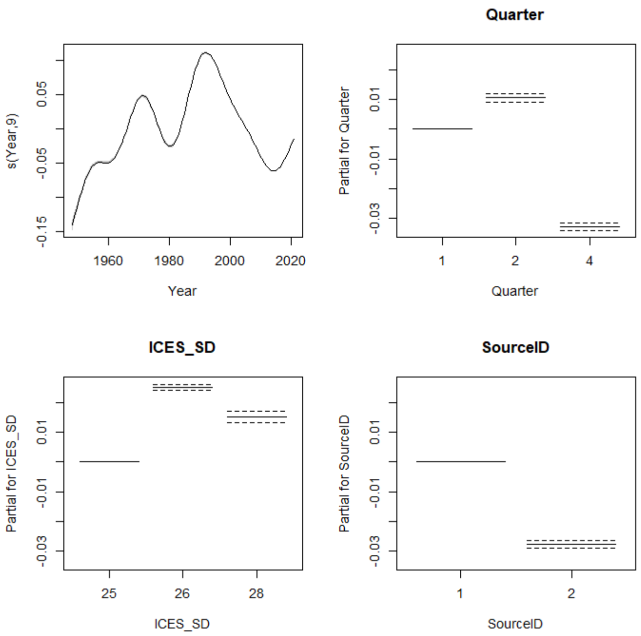

Fig B. Results of GAM analyses of cod body condition. The partial effects of quarter, ICES Subdivision and data source (SourceID) on cod condition are shown. SourceID 1 and 2 refer to data from commercial catch sampling and from research surveys, respectively.
