# Supplementary material for: New insights into the recent collapse of Eastern Baltic cod from historical data on stock health
Source: PLoS One. 2023 May 25;18(5):e0286247. doi: 10.1371/journal.pone.0286247 (PMC10212152; doi:10.1371/journal.pone.0286247)
Supplement: S2 Appendix — (PDF) [file pone.0286247.s004.pdf]

S2 Appendix. Sensitivity analyses of L95 indicator.

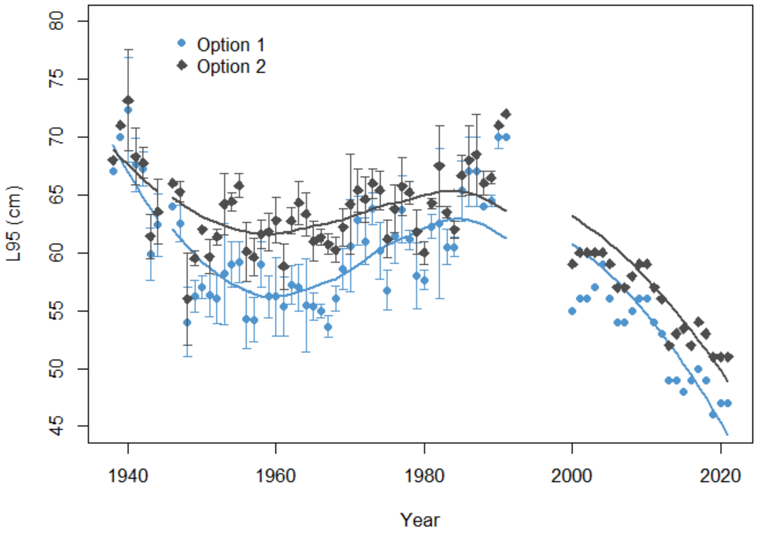

Fig A. Comparison of L95 indicators, estimated from entire length range (Option 1) and including only fish >40cm in length (Option 2). Based on length structure of commercial catches. The bars show standard error of the mean L95, in years where several datasets of length structure were available. The lines illustrate smoothed trends over time.

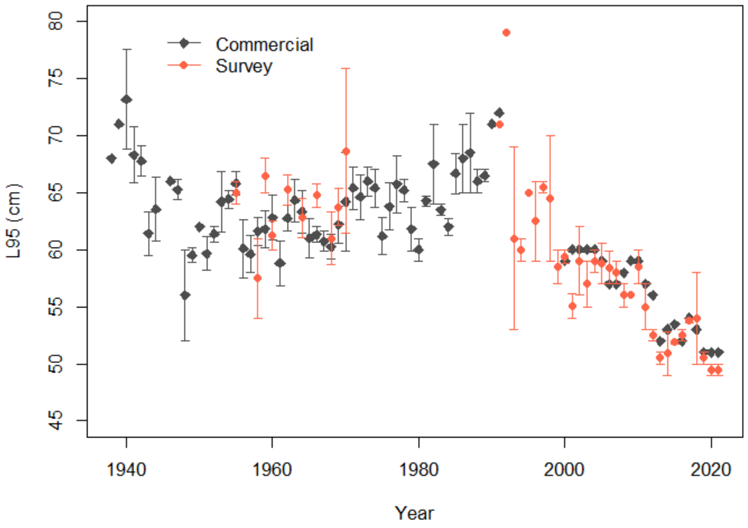

Fig B. Comparison of L95 indicators estimated from commercial and survey data. The calculation included only fish >40cm in length. The bars show standard error of the mean L95, in years where several length structure datasets were available.
