# Supplementary material for: New insights into the recent collapse of Eastern Baltic cod from historical data on stock health
Source: PLoS One. 2023 May 25;18(5):e0286247. doi: 10.1371/journal.pone.0286247 (PMC10212152; doi:10.1371/journal.pone.0286247)
Supplement: S3 Appendix — (PDF) [file pone.0286247.s005.pdf]

S3 Appendix. Data sources and sampling sizes for indicators of Eastern Baltic cod stock health

Table A. Numbers of individual cod (n) included in the analyses of body condition, hepatosomatic index (HSI), length at maturity (L50), and sex ratio (SR). The data sources for years prior to 1991 refer to archives of the research institutes in Germany (DE– Thünen Institute of Baltic Sea fisheries); Latvia (LV- Institute of Food Safety, Animal Health and Environment (BIOR)); Poland (POL– Polish National Marine Fisheries Research Institute) and Denmark (DK – Technical university of Denmark, Danish National Institute of Aquatic Resources). BITS– Baltic International Trawl Survey.

Year	n-condition	Source	n-HSI	Source	n- L50	Source	n-SR	Source
1948	468	LV			470	LV	270	LV
1949	159	LV			159	LV	119	LV
1950	952	LV			1558	LV	1051	LV
1951	581	LV			619	LV	368	LV
1952	346	LV					896	LV,DE
1953	165	DE	70	DE	517	LV	370	LV,DE
1954	2064	LV,DE	697	DE	2348	LV,DE	974	LV,DE
1955	1749	LV,DE	233	DE	2129	LV,DE	1138	LV,DE
1956	1648	LV,DE			1374	LV	982	LV,DE
1957	1796	LV,DE			1533	LV, DE	404	LV,DE
1958	1822	LV,DE					339	LV,DE
1959	3169	LV,DE			3180	LV,DE	962	LV,DE
1960	3416	LV,DE			3431	LV,DE	1363	LV,DE
1961	2473	LV,DE			2522	LV,DE	764	LV,DE
1962	872	LV,DE			871	LV,DE	267	LV,DE
1963	1889	LV			1916	LV	746	LV
1964	1819	LV			2011	LV	1339	LV
1965	3392	LV			3472	LV	1159	LV
1966	1653	LV			1672	LV	386	LV
1967	1598	DE, POL			1619	DE, POL	676	DE, POL
1968	2473	DE, POL	78	LV	1240	DE	820	DE
1969	2872	DE, POL			842	DE	389	DE
1970	3409	DE, POL	289	LV	1845	DE, POL	1104	DE, POL
1971	2632	DE, POL					363	DE
1972	2943	DE, POL	173	DE, LV	850	DE	626	DE
1973	1766	DE, POL	165	DE, LV	1780	DE, POL	1375	DE, POL
1974	245	DE	130	LV	249	DE		
1975	535	DE	93	LV	1150	DE, POL	584	DE, POL
1976	1798	DE, LV	107	LV	1808	DE, LV	1419	DE, LV
1977	2458	DE, POL, LV	66	LV	2280	DE, POL, LV	1940	DE, POL, LV
1978	959	DE, LV	90	LV	892	LV	601	DE, LV
1979	1339	DE, LV	37	LV	912	LV	456	DE, LV
1980	1829	LV			1830	LV	709	LV
1981	940	LV	391	LV	1012	LV	710	LV
1982	3452	LV	456	LV	3585	LV	1364	LV
1983	2932	POL, LV,DK	301	LV	2669	POL, LV	1605	DE,POL, LV
1984	925	LV	351	LV	925	LV	1059	DE, LV
1985	2864	DE, LV, POL	516	LV	2194	POL, LV	1997	DE,POL, LV
1986	1206	DE, LV	205	LV	731	LV	883	DE, LV
1987	1154	POL, LV,DK	87	LV	1020	POL, LV	747	POL, LV

1988	2587	POL, LV, DE, DK	298	LV	2599	DE,POL, LV	1402	DE,POL, LV
1989	2459	POL, DE, LV	374	LV	2103	DE,POL, LV	1886	DE,POL, LV
1990	2478	POL, LV	232	LV	2483	POL, LV	1744	POL, LV
1991	1894	BITS, DE			2080	BITS	994	BITS
1992	883	BITS, DE			439	BITS	119	BITS
1993	3183	BITS, DE			1364	BITS	1060	BITS
1994	5156	BITS, DE			1866	BITS	2336	BITS
1995	11420	BITS, DK, DE			2223	BITS	5149	BITS
1996	8731	BITS, DK, DE			2904	BITS	3341	BITS
1997	7034	BITS, DK			3919	BITS	2590	BITS
1998	6118	BITS, DK, DE	421	DK	4492	BITS	2173	BITS
1999	6040	BITS, DK			5041	BITS	2332	BITS
2000	7644	BITS, DK, DE			4208	BITS	2200	BITS
2001	4226	BITS, DK, DE			1214	BITS	767	BITS
2002	7097	BITS, DK, DE	23	DK	3505	BITS	1320	BITS
2003	8912	BITS, DK, DE	27	DK	3717	BITS	2337	BITS
2004	12727	BITS, DK, DE	754	DK	6207	BITS	3096	BITS
2005	13217	BITS, DK, DE	904	DK, DE	5965	BITS	3630	BITS
2006	12337	BITS, DK, DE	881	DK, DE	4863	BITS	3354	BITS
2007	13283	BITS, DK, DE	1247	DK, DE	5591	BITS	4244	BITS
2008	16646	BITS, DK, DE	1400	DK, DE	6844	BITS	4491	BITS
2009	14588	BITS, DK, DE	259	DK	5242	BITS	3002	BITS
2010	15246	BITS, DK, DE	1040	DK, DE	5268	BITS	3858	BITS
2011	11742	BITS, DK, DE	632	DK, DE	5031	BITS	2668	BITS
2012	7546	BITS, DK, DE	900	DK, DE	2559	BITS	1371	BITS
2013	9289	BITS, DK, DE	389	DK, DE	4756	BITS	1553	BITS
2014	6382	BITS, DK, DE	349	DK, DE	2713	BITS	1502	BITS
2015	7080	BITS, DK, DE	595	DK, DE	2532	BITS	1526	BITS
2016	7403	BITS, DE	504	DK, DE	2293	BITS	2025	BITS
2017	6437	BITS, DE	553	DK, DE	3016	BITS	1712	BITS
2018	5688	BITS, DE	308	DK, DE	3182	BITS	1071	BITS
2019	4565	BITS, DE	345	DK, DE	2868	BITS	724	BITS
2020	4383	BITS, DE	305	DK, DE	2813	BITS	776	BITS
2021	4954	BITS, DE	34	DE	3123	BITS	738	BITS

Table B. Data sources for spatially disaggregated commercial landings of the Eastern Baltic cod, by country.

Country	Years	Source
Denmark	1946–1964	National report series “Fiskeriberetning” published by the Danish Ministry of Agriculture (later the Ministry of Fisheries)
	1965–1971	[1]
Finland	1953–1971	ICES Bulletin Statistique des Peches Maritimes
German Fed. Rep.	1946–1962	[2]
	1963–1971	[3]
German De. Rep.	1949–1962	[4, 5, 6, 7, 8 and H. Müller pers. comm.]
	1963–1971	[9, 10]
Poland	1946–1960	[11]
	1961–1971	[12]

Sweden	1946–1959	National report series series FISKE published by the Royal Statistics Central Bureau [13]
	1960–1971	[3, 10, 12]
USSR	1946–1959	Total catch in these years [14, 15, M. Plikshs pers. comm] is divided to Subdivisions applying average catch distribution in 1960-1970
	1960–1972	[16]
All	1972–2021	[17]

Table C. Data sources for parasitic liver worm (*Contraceacum osculatum*) infection loads in Eastern Baltic cod. The table shows also the years and areas of sampling, including approximate ICES Subdivision (SD), and numbers (n) and length range of investigated cod.

Year of sampling	Sampling areas	SD	n	Cod length (cm)	Source
1946	Liepaja	26, 28	25	35-73	[18]
1949	Gotland trench, Liepaja	26, 28	742	30-100	[19]
1950,1955	Klaipeda	26	1000	45-60	[20]
1974–1975	Pomeranian Bay and adjacent waters	24, 25	1215	33-90	[21]
1982–1983	Bornholm Basin	25	92	30-100	[22]
1987–1990	Polish, Danish, Swedish waters	25, 26	2816	>31	[23]
2012	Bornholm Basin	25	185	30-80	[22]
2013–2015	Bornholm Basin	25	100	31-50	[24]
2016–2017	Bornholm Basin, south of Gotland	25	321	35-50	[25]
2017, 2019	Bornholm Basin	25	304	30-53	[26]
2017–2020	Bornholm Basin	25	370	30-58	[27]

Table D. Data sources for length compositions of Eastern Baltic cod.

(i) The historical data on length compositions of commercial catches, by countries. The data from 2000 onwards were for the total catch. The data "reported to ICES" refers to the materials that countries historically had provided to ICES stock assessment working groups in paper form (O. Bagge and E. Ojaveer pers. comm).

Years	German Fed. Rep.	German Dem. Rep.	Poland	USSR	Total
1938–1944	[28]				
1946				[29]	
1947	[28]		[30]	reported to ICES	
1948–1951			[31, 32]	reported to ICES	
1952–1960		H. Müller, pers. comm.	[32, 33, 34,35]	reported to ICES	
1961–1962		H. Müller pers. comm.		reported to ICES	
1963–1964				reported to ICES	
1965–1966			[36, 37]	reported to ICES	
1967–1968		reported to ICES	[36, 38]	reported to ICES	
1969–1978	reported to ICES	reported to ICES	[30, 38, 39, 40, 41, 42, 43, 44, 45, 46]	reported to ICES	

1979–1980	reported to ICES		[47, 48]		
1981	reported to ICES		reported to ICES	reported to ICES	
1982–1983	reported to ICES			reported to ICES	
1984–1985	reported to ICES		reported to ICES	reported to ICES	
1986–1990	reported to ICES		reported to ICES		
1991			reported to ICES		
2000–2021					[17]

(ii) Data sources for cod length compositions from research surveys.

**Years**	**Nation**	**Source**
1955–1960	USSR	[49, 50]
1962–1970	German Fed. Rep.	[51]
1991–2021	International (BITS)	ICES DATRAS Database

References

1. ICES. Report of the Working Group on Assessment of Demersal Stocks in the Baltic. ICES Document CM. 1977; P: 2. 17 pp.
2. Kändler R, Thurow F. On the stock of flatfish and cod and the yields of the German fishery in the Baltic. Rapp. Et Proc. Verd. 1959; 147.
3. ICES. Report of the Working Group on Assessment of Demersal Stocks in the Baltic. ICES Document CM. 1979; J: 4. 36 pp.
4. Berner M. 1959. Die Saßnitzer Dorscherträge der Jahre 1950-1958 und die Fangaussichten 1959 in ihrer Abhängigkeit von biologischen,hydrographischen und fischereilichen Faktoren. Fischereiforschung. 1959; 4/5: 5–8 (in German).
5. Berner M. Untersuchungen über den Dorschbestand (*Gadus morhua* L.) der Bornholm und Arkonasee in den Jahren1953-1955. Zeitschrift für Fischerei und deren Hilfswissenschaften N. F. 1960; IX (7-10): 481–602 (in German).
6. Berner M. Die Ostseedorscherträge 1960/61 und die Fangsituation 1962. Deutsche Fischerei-Zeitung. 1962; IX(4): 126–131 (in German).
7. Berner M, Anwand K. Die Fangplätze der südlichen Ostsee und ihre fischereiliche Bedeutung 1956-1960 im Vergleich zu 1953-1955. Zeitschrift für Fischerei. 1962/1963; 9 (1/2): 79–113 (in German).
8. Borrmann H, Berner M. Gesamtfänge der See- und Küstenfischerei der DDR aus der Ostsee in den Jahren 1947-1962. Fischerei-Forschung. 1984; 22(3): 11–22 (in German).
9. Berner M, Borrmann H. Development of the Baltic Cod Stock in ICES Subregion SD 25-32 (East of Bornholm) during 1965-1976. Fischerei-Forschung. 1980; 18(2):11–23 (in German).
10. ICES. Report of the Working Group on Assessment of Demersal Stocks in the Baltic. ICES Document CM. 1975; P: 17. 15 pp.
11. Rutkowicz S. The results of investigations on the stock of cod (*Gadus callarias*) in the south-eastern area of the Baltic sea in the years 1946–1960. Pr. Morsk. Inst. Ryb. Gdyni. 1963; 12/A:165–211.
12. ICES. Report of the Working Group on Assessment of Demersal Stocks in the Baltic. ICES Document CM. 1974; F: 4. 20 pp.

13. Awebro K, Poulsen B, editors. 2002. Swedish Baltic Catch data, 1752–1990. In Barnard MG, Nicholls JH (Comp.) HMAP Data Pages (www.hull.ac.uk/hmap).
14. Birjukov NP, Seletskaja AV, Tokareva GI. Sostojanie zapasov osnovnyh promyshlovyh ryb v tsentralnoi tsastjah Baltiskova morja i perspektivy ih promysla. Trydy BaltNIRO. 1960; 6 (in Russian).
15. Birjukov NP. Predislovie. Zapasy i biologia promyslovyh ryb juzhnoi tsasti Baltiiskova morja i evo limanov. AtlantNIRO. 1969 (in Russian).
16. Lablaika, IA, Lishev MV, Uzars DV, Chozjojski SA. Struktur, Zustand und Nutzung der Vorkommen des Ostseedorsches und seine Badetung in der Nahrungskette. Fischerei-forschung. 1975; 1 Sonderheft:31–37 (in German).
17. ICES. Baltic Fisheries Assessment Working Group (WGBFAS). ICES Scientific Reports. 2021; 3:53. 717 pp. https://doi.org/10.17895/ices.pub.8187
18. Shulman SS. A helminth disease of the liver of cod. Rybnoe Khozyaistvo, Leningrad. 1948; 4:38–40 (in Russian).
19. Petrushevsky GK, Shulman SS. Infection of the liver of Baltic cod with roundworms. Tr. Akad. Nauk. Litov. SSR. 1955; Ser. B 2: 119–125 (in Russian).
20. Gecevicjute S. Seasonal infection of the Baltic cod liver with *Contracaecum aduncum*. Trudy A.N. Litovskoj SSR, Serija B. 1955; 2:127–129 (in Russian).
21. Grabda J. The occurrence of anisakid nematode larvae in Baltic cod *(Gadus morhua* L.) and the dynamics of their invasion. Acta. Ichthyol. Piscat*.* 1976*;* 6 (1): 3–22 .
22. Haarder S, Kania PW, Galatius A, Buchmann K. Increased *contracaecum osculatum* infection in Baltic cod (*Gadus morhua*) livers (1982–2012) associated with increasing grey seal (*Halichoerus gryphus*) populations. J. Wildl. Dis. 2014; 50: 537–543.
23. Myjak P, Szostakowska B, Wojciechowski J, Pietkiewicz H, Rokicki J. Anisakid larvae in cod from the southern Baltic Sea. Arch. Fish. Mar. Res. 1994*;* 42(2): 146–161.
24. Zuo S, Huwer B, Bahlool Q, Al-Jubury A, Christensen ND, Korbut R, Kania P, Buchmann K. Host size-dependent anisakid infection in Baltic cod *Gadus morhua* associated with differential food preferences. Dis. Aquat. Organ. 2016; 120: 69–75.
25. Sokolova M, Buchmann K, Huwer B, Kania PW, Krumme U, Galatius A, Hemmer-Hansen J, Behrens JW. Spatial patterns in infection of cod *Gadus morhua* with the seal-associated liver worm *Contracaecum osculatum* from the Skagerrak to the Central Baltic Sea. Mar. Ecol. Prog. Ser. 2018; 606: 105–118.
26. Ryberg MP, Skov PV, Vendramin N, Buchmann K, Nielsen A, Behrens JW. 2020. Physiological condition of Eastern Baltic cod, *Gadus morhua*, infected with the parasitic nematode *Contracaecum osculatum*. Conserv. Physiol. 2020; 8(1): coaa093. doi:10.1093/conphys/coaa093
27. Ryberg MP, Huwer B, Nielsen A, Dierking J, Buchmann K, Sokolova M, et al. Parasite load of Atlantic cod *Gadus morhua* in the Baltic Sea assessed by the liver category method, and associations with infection density and critical condition. Fisheries Management and Ecology. 2022; 29(1):88–99.
28. Meyer PF. Die Dampferfischerei in der ostsee während der Kriegsjahre 1939/45 und ihre Bedeutung für die Fischwirtschaft und Fischereiwissenschaft. Ber. Dt. Wiss. Kommn. Meeresforsch. 1951; 12(1):168–209 (in German).
29. Dementjeva TF. Some data on the life history and fishery of cod in the central Baltic. Rapp. P.-v. Reun. Cons. Int. Explor. Mer. 1959; 147: 68–73.
30. Chrzan F. 1950. Investigations on the Baltic Cod. Journal du Conseil. 1950; 16:2.
31. Chrzan F. 1954. Investigations on the stock composition of cod-catches in the southern-eastern Baltic in 1948-1951 period. Rep. Sea. Fish. Inst. Gdynia. 1954; 7 (in Polish).
32. Zhukowski C. Investigations on the cod in the southern part of the Bornholm Basin in the years 1951–1952. Prace MIR. Inst. Ryb. 1957; 9.
33. Reimann Z. Composition of cod catches of the Bornholm region in 1953–1954. Prace. Morsk. Inst. Ryb. 1962; 11/A.

34. Stanek E. The stock of cod in the southern part of the Bornholm Basin in the years 1955–56. Pr. Morsk. Inst. Ryb. Gdyni. 1962; 11/A.
35. Stanek E. Biological characteristic of cod from Bornholm Basin in 1957–1960. Prace Morsk. Inst. Ryb. 1965; 3:57–85.
36. Kosior M. Polish cod investigations in the southern Baltic in the years 1966 and 1967. Annales Biol. Copenh. 1967; 24:101–102.
37. Kosior M. Cod of the southern Baltic in the years 1965-1967. Prace Morsk. Inst. Ryb. 1971; 16/A.
38. Kosior M. Polish cod investigations in the southern Baltic in 1968 and 1969. Annales Biol. 1969; 26:130–131.
39. Kosior M. Polish cod investigations in the southern Baltic in 1970. Annals Biol. 1970; 27:90.
40. Kosior M. Polish investigations in the southern Baltic in 1971. Annales Biol. 1971; 28:102–103.
41. Kosior M. Polish cod investigations in the southern Baltic in 1972. Annales Biol. 1972; 29:81.
42. Kosior M. Polish cod investigations in the southern Baltic in 1973. Annales Biol. 1973; 30:94–95.
43. Kosior M. Polish investigations on the state of the southern Baltic cod stocks in 1974, Annales Biol. 1974; 31:91–92.
44. Kosior M. Polish investigations on Southern Baltic cod stocks in 1975 and 1976. Annales. Biol. 1976; 33:90–91.
45. Kosior M. Polish investigations on the state of the Southern Baltic Baltic cod stocks in 1977, Annales. Biol. 1977; 34:121–122.
46. Kosior M. Polish investigations on southern Baltic cod stocks in 1978. Annales. Biol. 1978; 35: 134–135.
47. Kosior M. Polish investigations of southern Baltic cod stocks in 1979. Annales. Biol. 1979; 36:111–112.
48. Kosior M. Polish investigations on Southern Baltic cod stocks in 1980. Annales. Biol. 1980; 37:133–134.
49. Birjukov NP. 1962. Otsenka sostojanija zapasov treski baltiskova morja v 1960 i prognoz vozmozchnova jeje vylova v 1962. Trudy BaltNIRO. 1962; 8 (in Russian).
50. Tokareva G, Prieditis A. Russian investigations in the Eastern Baltic. Annales Biol. 1955; 12:142.
51. Tiews K. Further results of studies on the spawning stock of cod in the middle Baltic Sea. Rapp. P.-v. Reun. Cons. Int. Explor. Mer. 1971; 166:66–82.
