# Supplementary material for: New insights into the recent collapse of Eastern Baltic cod from historical data on stock health
Source: PLoS One. 2023 May 25;18(5):e0286247. doi: 10.1371/journal.pone.0286247 (PMC10212152; doi:10.1371/journal.pone.0286247)
Supplement: S4 Appendix — (PDF) [file pone.0286247.s006.pdf]

S4 Appendix. Data for indicators of Eastern Baltic cod stock health

Table A. Time series of indicators of Eastern Baltic cod stock health. The data shown are mean values for the following indicators: body condition (LeCren's *K*); length at first maturity (L50; in cm); sex ratio (SR), represented as proportion of females among >40 cm cod and >50 cm cod; distribution, represented as fraction of total eastern Baltic cod landings taken in ICES Subdivisions 27–32 (bars), i.e. in the northernmost edge of distribution range of the stock; length at 95th percentile of length distribution (L95; in cm). Standard error of the mean (se) is shown where appropriate.

Year	K	se_K	L50	se_L50	SR (40cm)	SR (50cm)	Distribution	L95	se_L95
1938	-	-	-	-	-	-	-	68.0	-
1939	-	-	-	-	-	-	-	71.0	-
1940	-	-	-	-	-	-	-	73.2	4.347
1941	-	-	-	-	-	-	-	68.3	2.437
1942	-	-	-	-	-	-	-	67.8	1.315
1943	-	-	-	-	-	-	-	61.4	1.901
1944	-	-	-	-	-	-	-	63.6	2.775
1945	-	-	-	-	-	-	-	-	-
1946	-	-	-	-	-	-	0.05	66.0	-
1947	-	-	-	-	-	-	0.05	65.3	0.901
1948	0.95	0.008	31.9	1.106	0.67	0.73	0.04	56.0	4.000
1949	0.92	0.010	32.0	1.405	0.31	0.45	0.05	59.5	0.645
1950	0.89	0.005	33.3	0.600	0.55	0.67	0.08	62.0	0.000
1951	1.00	0.005	39.9	0.618	0.70	0.84	0.08	59.7	1.453
1952	0.88	0.007	-	-	0.53	0.65	0.08	61.4	0.687
1953	0.95	0.009	30.6	0.714	0.56	0.63	0.07	64.2	2.653
1954	0.94	0.003	32.0	0.467	0.59	0.63	0.08	64.4	0.748
1955	0.95	0.003	34.5	0.333	0.55	0.68	0.09	65.8	1.020
1956	0.98	0.003	31.7	0.633	0.53	0.60	0.10	60.1	2.544
1957	1.03	0.003	32.0	0.811	0.52	0.55	0.08	59.6	1.631
1958	0.99	0.003	-	-	0.60	0.57	0.09	61.6	1.208
1959	1.02	0.003	37.5	0.214	0.62	0.69	0.08	61.8	1.655
1960	0.88	0.002	37.7	0.247	0.42	0.53	0.06	62.8	1.985
1961	1.01	0.003	39.1	0.266	0.57	0.70	0.05	58.8	1.956
1962	0.96	0.004	30.7	1.077	0.40	0.32	0.07	62.8	1.109
1963	1.06	0.004	40.6	0.389	0.68	0.80	0.04	64.3	1.856
1964	1.05	0.004	32.9	0.543	0.59	0.73	0.03	63.3	1.856
1965	1.00	0.003	35.4	0.255	0.51	0.69	0.05	61.0	1.732
1966	1.00	0.005	37.5	0.364	0.46	0.53	0.11	61.3	0.667
1967	1.03	0.003	29.5	0.666	0.49	0.53	0.07	60.8	0.854
1968	1.07	0.002	32.8	0.444	0.53	0.62	0.07	60.3	1.109
1969	1.07	0.002	34.6	0.463	0.50	0.57	0.04	62.2	1.655
1970	1.09	0.002	31.5	0.464	0.58	0.71	0.04	64.2	4.329
1971	1.08	0.002	-	-	0.56	0.72	0.05	65.4	1.887
1972	1.08	0.003	32.9	0.754	0.56	0.58	0.05	64.6	1.990
1973	1.11	0.004	33.7	0.487	0.57	0.69	0.10	66.0	1.265
1974	1.07	0.007	39.8	0.847	-	-	0.15	65.4	1.691
1975	1.04	0.005	29.9	0.719	0.57	0.71	0.18	61.2	1.594
1976	1.05	0.003	28.8	0.658	0.33	0.48	0.10	63.8	2.083
1977	1.09	0.003	33.7	0.498	0.46	0.59	0.11	65.8	2.496
1978	0.97	0.004	33.6	0.558	0.34	0.46	0.17	65.2	0.970
1979	0.98	0.004	38.8	0.671	0.55	0.58	0.24	61.8	1.908
1980	1.00	0.003	41.8	0.355	0.59	0.69	0.27	60.0	1.000
1981	0.97	0.004	41.6	0.374	0.59	0.66	0.19	64.3	0.479
1982	1.01	0.002	43.3	0.190	0.53	0.63	0.25	67.5	3.500
1983	1.02	0.003	42.2	0.225	0.56	0.66	0.26	63.5	0.500
1984	1.01	0.004	37.0	0.581	0.54	0.61	0.24	62.0	0.707
1985	1.03	0.003	41.8	0.237	0.56	0.65	0.23	66.7	1.764
1986	1.05	0.004	37.9	0.610	0.53	0.64	0.21	68.0	3.000
1987	1.09	0.004	39.1	0.447	0.51	0.59	0.20	68.5	3.500
1988	1.11	0.003	39.3	0.267	0.61	0.70	0.18	66.0	1.000
1989	1.10	0.003	35.8	0.540	0.56	0.64	0.13	66.5	0.500
1990	1.16	0.003	37.2	0.318	0.58	0.64	0.12	71.0	0.000
1991	1.18	0.004	39.4	0.304	0.57	0.64	0.11	72.0	-
1992	1.14	0.004	32.7	0.563	0.59	0.70	0.11	-	-

1993	1.11	0.003	37.0	0.398	0.58	0.70	0.09	-	-
1994	1.08	0.002	33.5	0.294	0.63	0.73	0.09	-	-
1995	1.11	0.001	37.9	0.138	0.59	0.73	0.07	-	-
1996	1.10	0.002	39.0	0.212	0.60	0.69	0.06	-	-
1997	1.09	0.002	39.6	0.238	0.58	0.64	0.05	-	-
1998	1.05	0.001	37.5	0.221	0.53	0.61	0.05	-	-
1999	1.04	0.001	35.0	0.194	0.57	0.68	0.05	-	-
2000	1.03	0.001	33.6	0.192	0.60	0.69	0.04	59.0	-
2001	1.04	0.002	31.6	0.326	0.64	0.70	0.04	60.0	-
2002	1.04	0.002	31.1	0.212	0.63	0.71	0.02	60.0	-
2003	1.05	0.002	32.0	0.224	0.62	0.71	0.00	60.0	-
2004	1.02	0.001	30.8	0.171	0.59	0.71	0.02	60.0	-
2005	1.00	0.001	30.7	0.204	0.62	0.70	0.01	59.0	-
2006	1.00	0.002	28.0	0.271	0.61	0.67	0.01	57.0	-
2007	1.00	0.001	29.2	0.191	0.63	0.70	0.01	57.0	-
2008	0.98	0.001	27.4	0.184	0.64	0.73	0.01	58.0	-
2009	0.97	0.001	25.9	0.282	0.68	0.75	0.01	59.0	-
2010	0.97	0.001	26.8	0.243	0.65	0.74	0.00	59.0	-
2011	0.95	0.001	27.3	0.305	0.68	0.82	0.00	57.0	-
2012	0.95	0.001	27.1	0.367	0.66	0.75	0.01	56.0	-
2013	0.94	0.001	24.6	0.237	0.65	0.74	0.01	52.0	-
2014	0.95	0.001	26.5	0.554	0.69	0.81	0.01	53.0	-
2015	0.96	0.002	22.0	0.681	0.73	0.79	0.01	53.4	-
2016	0.93	0.002	20.6	0.488	0.68	0.84	0.01	52.0	-
2017	0.95	0.002	20.7	0.384	0.66	0.76	0.03	54.0	-
2018	0.95	0.002	20.6	0.513	0.67	0.87	0.01	53.0	-
2019	0.98	0.002	20.6	0.513	0.69	0.76	0.02	51.0	-
2020	0.98	0.002	20.2	0.425	0.68	0.76	0.02	51.0	-
2021	0.96	0.002	16.4	0.610	0.61	0.82	0.03	51.0	-

Table B. Data for body condition (Le Cren's K) of the Eastern Baltic cod. Data are shown separately for quarters and ICES Subdivisions. Mean values and standard error of the mean (se) are shown.

Year	Quarter	ICES_SD	LeCren's K	se
1948	1	26	0.89	0.006
1948	2	28	1.14	0.020
1949	2	26	0.92	0.010
1950	1	26	0.91	0.015
1950	1	28	0.88	0.010
1950	2	26	0.94	0.006
1950	2	28	0.83	0.008
1951	1	26	1.01	0.007
1951	2	26	0.99	0.008
1952	1	28	0.85	0.007
1952	2	28	0.96	0.014
1953	2	25	0.94	0.018
1953	4	25	0.95	0.010
1954	1	25	0.92	0.005
1954	1	26	1.01	0.012
1954	2	25	0.92	0.004
1954	2	26	0.92	0.014
1954	2	28	0.85	0.007
1954	4	25	1.01	0.007
1955	1	25	0.93	0.009
1955	1	26	0.97	0.005
1955	1	28	0.94	0.008
1955	2	25	0.94	0.003
1956	1	25	0.95	0.009
1956	1	26	0.99	0.007
1956	2	25	1.00	0.004
1956	2	26	0.97	0.010
1956	2	28	0.93	0.009
1957	1	25	1.04	0.007
1957	1	26	1.01	0.015
1957	2	25	1.06	0.005
1957	2	26	1.01	0.010

1957	2	28	0.93	0.013
1957	4	25	0.99	0.008
1957	4	26	0.95	0.016
1958	1	25	1.06	0.005
1958	1	28	0.98	0.009
1958	2	28	0.95	0.006
1958	4	25	0.98	0.005
1959	1	25	1.01	0.004
1959	1	26	0.86	0.013
1959	1	28	0.98	0.006
1959	2	25	0.93	0.007
1959	2	26	1.03	0.006
1959	2	28	1.11	0.006
1960	1	25	0.94	0.006
1960	1	26	0.85	0.003
1960	1	28	0.84	0.005
1960	2	25	1.09	0.011
1960	2	26	0.89	0.005
1960	2	28	0.86	0.007
1961	1	25	0.98	0.005
1961	1	26	0.96	0.006
1961	1	28	1.06	0.011
1961	2	25	1.00	0.011
1961	2	26	1.05	0.007
1961	2	28	0.99	0.010
1962	1	25	0.98	0.006
1962	2	25	1.00	0.010
1962	2	26	0.94	0.006
1963	1	26	1.17	0.014
1963	1	28	1.18	0.015
1963	2	25	1.04	0.006
1963	2	26	1.02	0.007
1963	2	28	0.98	0.014
1964	1	25	1.00	0.017
1964	1	26	0.96	0.016
1964	2	26	1.06	0.004
1964	2	28	1.12	0.061
1965	1	25	0.95	0.009
1965	1	26	0.96	0.005
1965	1	28	0.98	0.060
1965	2	25	1.00	0.008
1965	2	26	1.02	0.005
1965	2	28	1.10	0.024
1966	1	28	0.96	0.011
1966	2	26	1.01	0.006
1966	2	28	0.98	0.014
1967	1	25	1.06	0.007
1967	1	26	1.03	0.004
1967	2	25	0.99	0.009
1967	2	26	1.03	0.005
1968	1	25	1.08	0.003
1968	1	26	1.09	0.004
1968	2	25	1.03	0.006
1968	2	26	1.07	0.013
1968	4	25	1.04	0.010
1969	1	25	1.06	0.003
1969	1	26	1.07	0.003
1970	1	25	1.07	0.003
1970	1	26	1.09	0.004
1970	2	25	1.11	0.006
1971	1	25	1.08	0.003
1971	1	26	1.09	0.004
1971	2	25	1.00	0.010
1972	1	25	1.09	0.003
1972	1	26	1.11	0.005
1972	2	26	1.00	0.007
1973	1	25	1.11	0.007
1973	1	26	1.15	0.005
1973	2	25	1.09	0.008
1973	2	26	1.09	0.009
1974	1	25	1.07	0.010

1974	1	26	1.07	0.010
1975	1	25	1.04	0.005
1976	1	26	1.08	0.006
1976	2	26	1.03	0.004
1977	1	26	1.12	0.004
1977	2	25	1.15	0.012
1977	2	26	1.09	0.005
1977	4	25	0.91	0.008
1978	1	26	0.99	0.006
1978	2	26	0.98	0.006
1978	4	25	0.93	0.010
1979	1	26	1.05	0.006
1979	1	28	0.96	0.009
1979	4	25	0.90	0.005
1980	1	26	0.99	0.007
1980	1	28	0.99	0.010
1980	2	26	1.01	0.006
1980	2	28	0.99	0.004
1981	1	26	0.97	0.005
1981	1	28	0.94	0.008
1981	2	26	0.98	0.007
1982	1	26	1.03	0.003
1982	1	28	1.01	0.004
1982	2	25	1.06	0.009
1982	2	26	1.01	0.004
1982	2	28	0.94	0.004
1983	1	25	0.89	0.005
1983	1	26	1.11	0.004
1983	1	28	0.98	0.004
1983	2	26	1.05	0.007
1983	2	28	0.95	0.005
1984	1	26	1.01	0.004
1985	1	26	1.09	0.005
1985	1	28	1.02	0.010
1985	2	26	1.00	0.007
1985	2	28	0.95	0.005
1985	4	25	1.00	0.006
1986	1	26	1.08	0.008
1986	2	26	1.05	0.007
1986	4	25	1.02	0.005
1987	1	25	0.96	0.009
1987	1	26	1.10	0.004
1988	1	25	1.00	0.006
1988	1	26	1.15	0.004
1988	1	28	1.07	0.004
1988	2	25	1.20	0.026
1989	1	25	0.98	0.007
1989	1	26	1.15	0.004
1989	1	28	1.10	0.014
1989	2	26	1.16	0.012
1989	2	28	1.10	0.009
1989	4	25	1.04	0.006
1990	1	25	1.13	0.007
1990	1	26	1.16	0.004
1990	1	28	1.20	0.013
1990	2	25	1.12	0.010
1990	2	26	1.16	0.006
1990	2	28	1.17	0.013
1991	1	25	1.05	0.008
1991	1	26	1.23	0.004
1991	1	28	1.14	0.010
1991	2	26	1.16	0.015
1991	4	25	1.08	0.009
1991	4	26	1.18	0.028
1991	4	28	1.07	0.024
1992	1	25	1.13	0.005
1992	1	26	1.18	0.011
1992	1	28	1.11	0.019
1993	1	25	1.04	0.003
1993	1	26	1.20	0.004
1993	1	28	1.08	0.014

1993	2	26	1.19	0.007
1993	2	28	1.18	0.034
1993	4	25	1.11	0.009
1993	4	28	1.22	0.025
1994	1	25	1.04	0.003
1994	1	26	1.10	0.007
1994	1	28	1.08	0.011
1994	2	26	1.19	0.005
1994	2	28	1.19	0.019
1994	4	25	1.06	0.005
1994	4	26	1.09	0.016
1994	4	28	1.07	0.013
1995	1	25	1.10	0.001
1995	1	26	1.16	0.002
1995	1	28	1.14	0.005
1995	4	25	1.04	0.004
1995	4	26	1.07	0.010
1995	4	28	1.09	0.008
1996	1	25	1.07	0.003
1996	1	26	1.11	0.004
1996	1	28	1.10	0.007
1996	2	25	1.09	0.006
1996	2	26	1.09	0.017
1996	4	25	1.19	0.005
1996	4	26	1.15	0.009
1996	4	28	1.22	0.034
1997	1	25	1.08	0.003
1997	1	26	1.12	0.003
1997	1	28	1.07	0.007
1997	2	25	1.09	0.004
1997	2	26	1.06	0.012
1997	2	28	1.07	0.021
1997	4	25	1.10	0.004
1997	4	26	1.02	0.007
1997	4	28	1.08	0.007
1998	1	25	1.05	0.002
1998	1	26	1.08	0.003
1998	1	28	1.05	0.005
1998	2	25	1.10	0.025
1998	2	28	0.96	0.009
1998	4	25	1.02	0.005
1998	4	26	1.07	0.010
1998	4	28	1.02	0.011
1999	1	25	1.03	0.002
1999	1	26	1.05	0.003
1999	1	28	1.03	0.006
1999	2	25	1.01	0.012
1999	2	28	1.01	0.012
1999	4	25	1.03	0.004
1999	4	26	1.02	0.009
1999	4	28	1.05	0.009
2000	1	25	1.02	0.002
2000	1	26	1.07	0.004
2000	1	28	1.03	0.004
2000	2	25	1.04	0.004
2000	2	28	1.14	0.030
2000	4	25	1.03	0.004
2000	4	26	1.04	0.004
2000	4	28	1.06	0.005
2001	1	25	1.03	0.004
2001	1	26	1.15	0.009
2001	1	28	1.03	0.007
2001	2	25	1.05	0.004
2001	4	25	1.01	0.004
2001	4	26	1.01	0.010
2001	4	28	1.03	0.009
2002	1	25	1.04	0.003
2002	1	26	1.02	0.004
2002	1	28	1.00	0.008
2002	2	25	1.06	0.003
2002	4	25	1.00	0.003

2002	4	26	1.14	0.008
2002	4	28	1.02	0.013
2003	1	25	1.04	0.003
2003	1	26	1.02	0.003
2003	1	28	0.99	0.008
2003	2	25	1.18	0.004
2003	4	25	1.02	0.004
2003	4	26	1.03	0.004
2003	4	28	1.03	0.008
2004	1	25	1.05	0.002
2004	1	26	1.02	0.002
2004	1	28	1.01	0.007
2004	2	25	1.06	0.003
2004	4	25	0.97	0.002
2004	4	26	0.98	0.004
2004	4	28	0.98	0.010
2005	1	25	1.01	0.002
2005	1	26	1.05	0.002
2005	1	28	1.02	0.006
2005	2	25	1.01	0.003
2005	4	25	0.95	0.002
2005	4	26	0.97	0.003
2005	4	28	0.95	0.006
2006	1	25	1.01	0.002
2006	1	26	1.02	0.006
2006	1	28	0.98	0.004
2006	2	25	1.00	0.003
2006	4	25	0.97	0.005
2006	4	26	0.98	0.004
2006	4	28	1.03	0.006
2007	1	25	1.01	0.002
2007	1	26	1.05	0.003
2007	1	28	1.01	0.005
2007	2	25	1.05	0.003
2007	4	25	0.96	0.002
2007	4	26	0.95	0.003
2007	4	28	1.04	0.006
2008	1	25	0.98	0.002
2008	1	26	1.03	0.002
2008	1	28	1.01	0.004
2008	2	25	1.04	0.003
2008	4	25	0.91	0.002
2008	4	26	0.93	0.003
2008	4	28	0.97	0.008
2009	1	25	0.95	0.002
2009	1	26	1.00	0.002
2009	1	28	0.95	0.005
2009	2	25	1.02	0.002
2009	4	25	0.92	0.002
2009	4	26	0.96	0.004
2009	4	28	0.97	0.006
2010	1	25	0.96	0.002
2010	1	26	0.98	0.002
2010	1	28	0.93	0.005
2010	2	25	0.99	0.002
2010	4	25	0.95	0.003
2010	4	26	0.94	0.003
2010	4	28	0.97	0.007
2011	1	25	0.95	0.002
2011	1	26	0.97	0.002
2011	1	28	0.92	0.006
2011	2	25	0.94	0.003
2011	4	25	0.93	0.004
2011	4	26	0.97	0.004
2011	4	28	0.99	0.009
2012	1	25	0.95	0.002
2012	1	26	0.97	0.003
2012	1	28	0.94	0.009
2012	2	25	0.93	0.003
2012	4	25	0.94	0.004
2012	4	26	0.94	0.006

2012	4	28	1.04	0.007
2013	1	25	0.96	0.003
2013	1	26	0.95	0.002
2013	1	28	0.94	0.008
2013	2	25	0.93	0.002
2013	4	25	0.93	0.004
2013	4	26	0.95	0.005
2013	4	28	0.96	0.006
2014	1	25	0.95	0.002
2014	1	26	0.98	0.005
2014	1	28	0.97	0.007
2014	2	25	0.99	0.004
2014	4	25	0.92	0.003
2014	4	26	0.93	0.003
2014	4	28	0.98	0.005
2015	1	25	0.97	0.003
2015	1	26	0.97	0.004
2015	1	28	0.97	0.006
2015	2	25	1.00	0.004
2015	4	25	0.91	0.003
2015	4	26	0.94	0.003
2015	4	28	1.02	0.007
2016	1	25	0.95	0.004
2016	1	26	0.96	0.004
2016	1	28	0.97	0.012
2016	2	25	1.00	0.005
2016	4	25	0.90	0.002
2016	4	26	0.93	0.003
2016	4	28	0.92	0.018
2017	1	25	0.98	0.002
2017	1	26	0.97	0.004
2017	1	28	0.93	0.005
2017	2	25	1.02	0.007
2017	4	25	0.90	0.003
2017	4	26	0.92	0.003
2017	4	28	1.01	0.010
2018	1	25	0.96	0.002
2018	1	26	0.98	0.003
2018	1	28	0.94	0.007
2018	2	25	0.93	0.006
2018	4	25	0.90	0.004
2018	4	26	0.91	0.005
2018	4	28	0.98	0.009
2019	1	25	1.01	0.002
2019	1	26	0.98	0.004
2019	1	28	0.98	0.005
2019	4	25	0.95	0.003
2019	4	26	0.94	0.005
2019	4	28	1.00	0.006
2020	1	25	1.03	0.003
2020	1	26	1.02	0.005
2020	1	28	0.98	0.006
2020	4	25	0.90	0.004
2020	4	26	0.94	0.005
2020	4	28	0.97	0.010
2021	1	25	0.97	0.003
2021	1	26	0.97	0.003
2021	1	28	0.96	0.005
2021	4	25	0.95	0.005
2021	4	26	0.91	0.005
2021	4	28	0.97	0.007

Table C. Data on Eastern Baltic cod landings by ICES Subdivisions (SD). See Table B in S3 Appendix for data sources.

Year	SD	Landings (1000 t)
1946	25	20.189
1947	25	31.688
1948	25	61.224
1949	25	59.894
1950	25	44.675
1951	25	52.339
1952	25	59.375
1953	25	50.970
1954	25	42.935
1955	25	46.825
1956	25	51.569
1957	25	75.860
1958	25	62.689
1959	25	60.529
1960	25	67.376
1961	25	62.386
1962	25	58.194
1963	25	63.337
1964	25	55.022
1965	25	57.119
1966	25	61.288
1967	25	73.378
1968	25	93.017
1969	25	93.746
1970	25	84.273
1971	25	67.079
1972	25	77.983
1973	25	92.854
1974	25	76.721
1975	25	100.002
1976	25	104.784
1977	25	78.710
1978	25	73.048
1979	25	99.657
1980	25	131.477
1981	25	153.609
1982	25	150.784
1983	25	167.433
1984	25	187.145
1985	25	144.219
1986	25	134.174
1987	25	109.633
1988	25	114.476
1989	25	122.851
1990	25	92.023
1991	25	76.088
1992	25	33.423
1993	25	20.371
1994	25	40.649
1995	25	61.554
1996	25	72.463
1997	25	58.144
1998	25	37.339
1999	25	43.702
2000	25	42.203
2001	25	43.334
2002	25	34.028
2003	25	32.423
2004	25	36.626
2005	25	36.336
2006	25	44.062
2007	25	34.726
2008	25	28.958
2009	25	33.073
2010	25	31.149
2011	25	29.596

2012	25	32.281
2013	25	15.673
2014	25	17.313
2015	25	22.612
2016	25	14.670
2017	25	10.388
2018	25	6.866
2019	25	3.339
2020	25	0.414
2021	25	0.106
1946	26	20.742
1947	26	38.392
1948	26	43.586
1949	26	49.917
1950	26	71.767
1951	26	77.313
1952	26	92.442
1953	26	61.587
1954	26	73.358
1955	26	60.237
1956	26	88.499
1957	26	93.891
1958	26	65.912
1959	26	59.170
1960	26	73.970
1961	26	49.730
1962	26	54.430
1963	26	60.150
1964	26	44.140
1965	26	39.493
1966	26	58.792
1967	26	67.993
1968	26	59.732
1969	26	68.970
1970	26	64.423
1971	26	45.156
1972	26	58.229
1973	26	36.660
1974	26	48.827
1975	26	59.054
1976	26	77.326
1977	26	67.209
1978	26	54.495
1979	26	73.884
1980	26	120.967
1981	26	115.992
1982	26	87.601
1983	26	79.732
1984	26	112.171
1985	26	96.981
1986	26	64.100
1987	26	56.124
1988	26	44.400
1989	26	31.858
1990	26	42.459
1991	26	31.658
1992	26	15.037
1993	26	8.490
1994	26	10.998
1995	26	19.542
1996	26	31.002
1997	26	24.663
1998	26	25.978
1999	26	24.070
2000	26	19.594
2001	26	19.890
2002	26	13.851
2003	26	13.308
2004	26	10.736
2005	26	17.684
2006	26	20.622

2007	26	15.308
2008	26	12.988
2009	26	15.064
2010	26	18.984
2011	26	20.559
2012	26	18.691
2013	26	15.416
2014	26	11.299
2015	26	15.120
2016	26	14.282
2017	26	14.098
2018	26	8.792
2019	26	4.854
2020	26	1.853
2021	26	1.234
1946	27	0.727
1947	27	0.922
1948	27	1.226
1949	27	1.458
1950	27	1.494
1951	27	1.675
1952	27	2.370
1953	27	2.519
1954	27	2.358
1955	27	2.522
1956	27	2.545
1957	27	2.893
1958	27	2.375
1959	27	2.578
1960	27	2.575
1961	27	2.163
1962	27	1.818
1963	27	1.515
1964	27	1.024
1965	27	0.624
1966	27	0.865
1967	27	0.839
1968	27	0.702
1969	27	0.514
1970	27	0.528
1971	27	0.833
1972	27	0.876
1973	27	0.972
1974	27	1.682
1975	27	2.055
1976	27	2.105
1977	27	2.588
1978	27	3.285
1979	27	3.458
1980	27	6.014
1981	27	7.200
1982	27	4.109
1983	27	6.490
1984	27	8.223
1985	27	7.068
1986	27	7.554
1987	27	5.708
1988	27	6.674
1989	27	7.703
1990	27	6.702
1991	27	5.104
1992	27	2.145
1993	27	0.940
1994	27	2.845
1995	27	2.181
1996	27	3.622
1997	27	2.417
1998	27	1.159
1999	27	1.866
2000	27	1.242
2001	27	1.683

2002	27	0.004
2003	27	0.002
2004	27	0.465
2005	27	0.104
2006	27	0.140
2007	27	0.145
2008	27	0.096
2009	27	0.079
2010	27	0.045
2011	27	0.041
2012	27	0.024
2013	27	0.027
2014	27	0.026
2015	27	0.031
2016	27	0.061
2017	27	0.097
2018	27	0.015
2019	27	0.011
2020	27	0.001
2021	27	0.001
1946	28	1.223
1947	28	2.675
1948	28	3.526
1949	28	4.889
1950	28	8.108
1951	28	9.270
1952	28	9.965
1953	28	5.940
1954	28	7.952
1955	28	7.822
1956	28	12.064
1957	28	12.473
1958	28	9.459
1959	28	8.234
1960	28	6.179
1961	28	3.767
1962	28	6.345
1963	28	3.619
1964	28	2.436
1965	28	4.833
1966	28	13.663
1967	28	10.131
1968	28	10.968
1969	28	6.591
1970	28	5.174
1971	28	5.050
1972	28	6.633
1973	28	12.465
1974	28	19.374
1975	28	31.288
1976	28	18.552
1977	28	15.651
1978	28	20.793
1979	28	42.598
1980	28	63.172
1981	28	36.645
1982	28	50.234
1983	28	48.802
1984	28	56.358
1985	28	38.630
1986	28	32.022
1987	28	20.758
1988	28	18.994
1989	28	12.890
1990	28	9.977
1991	28	7.551
1992	28	3.392
1993	28	1.552
1994	28	1.696
1995	28	3.632
1996	28	3.179

1997	28	1.828
1998	28	1.886
1999	28	1.892
2000	28	1.473
2001	28	1.191
2002	28	0.765
2003	28	0.048
2004	28	0.315
2005	28	0.624
2006	28	0.527
2007	28	0.392
2008	28	0.164
2009	28	0.192
2010	28	0.062
2011	28	0.088
2012	28	0.172
2013	28	0.183
2014	28	0.182
2015	28	0.091
2016	28	0.104
2017	28	0.633
2018	28	0.031
2019	28	0.052
2020	28	0.018
2021	28	0.002
1946	29	0.018
1947	29	0.005
1948	29	0.016
1949	29	0.000
1950	29	0.000
1951	29	0.000
1952	29	0.000
1953	29	0.000
1954	29	0.000
1955	29	0.000
1956	29	0.000
1957	29	0.000
1958	29	0.000
1959	29	0.000
1960	29	0.004
1961	29	0.000
1962	29	0.000
1963	29	0.001
1964	29	0.007
1965	29	0.003
1966	29	0.014
1967	29	0.000
1968	29	0.000
1969	29	0.000
1970	29	0.000
1971	29	0.000
1972	29	0.000
1973	29	0.050
1974	29	1.010
1975	29	2.128
1976	29	0.302
1977	29	0.303
1978	29	0.832
1979	29	2.924
1980	29	7.528
1981	29	7.481
1982	29	8.552
1983	29	12.677
1984	29	12.956
1985	29	14.583
1986	29	8.093
1987	29	3.946
1988	29	1.705
1989	29	1.164
1990	29	0.442
1991	29	0.413

1992	29	0.146
1993	29	0.051
1994	29	0.274
1995	29	0.059
1996	29	0.017
1997	29	0.016
1998	29	0.030
1999	29	0.010
2000	29	0.012
2001	29	0.018
2002	29	0.005
2003	29	0.000
2004	29	0.010
1946	30	0.122
1947	30	0.095
1948	30	0.060
1949	30	0.039
1950	30	0.052
1951	30	0.038
1952	30	0.082
1953	30	0.093
1954	30	0.070
1955	30	0.096
1956	30	0.085
1957	30	0.078
1958	30	0.087
1959	30	0.086
1960	30	0.063
1961	30	0.054
1962	30	0.066
1963	30	0.081
1964	30	0.026
1965	30	0.014
1966	30	0.019
1967	30	0.006
1968	30	0.002
1969	30	0.005
1970	30	0.001
1971	30	0.046
1972	30	0.112
1973	30	0.149
1974	30	0.201
1975	30	0.008
1976	30	0.029
1977	30	0.048
1978	30	0.378
1979	30	0.663
1980	30	1.396
1981	30	1.184
1982	30	3.037
1983	30	2.333
1984	30	3.012
1985	30	2.635
1986	30	1.604
1987	30	6.960
1988	30	6.713
1989	30	2.024
1990	30	1.038
1991	30	0.611
1992	30	0.475
1993	30	0.256
1994	30	0.147
1995	30	0.047
1996	30	0.006
1997	30	0.009
1998	30	0.004
1999	30	0.002
2000	30	0.001
2001	30	0.005
2002	30	0.000
2003	30	0.000

2004	30	0.004
1946	31	0.000
1947	31	0.000
1948	31	0.000
1949	31	0.000
1950	31	0.000
1951	31	0.000
1952	31	0.000
1953	31	0.000
1954	31	0.000
1955	31	0.000
1956	31	0.050
1957	31	0.000
1958	31	0.000
1959	31	0.000
1960	31	0.000
1961	31	0.000
1962	31	0.000
1963	31	0.000
1964	31	0.000
1965	31	0.000
1966	31	0.000
1967	31	0.000
1968	31	0.000
1969	31	0.000
1970	31	0.000
1971	31	0.000
1972	31	0.000
1973	31	0.000
1974	31	0.000
1975	31	0.000
1976	31	0.000
1977	31	0.000
1978	31	0.007
1979	31	0.022
1980	31	0.049
1981	31	0.019
1982	31	0.080
1983	31	0.036
1984	31	0.007
1985	31	0.030
1986	31	0.056
1987	31	0.039
1988	31	0.008
1989	31	0.032
1990	31	0.028
1991	31	0.012
1992	31	0.001
1993	31	0.002
1994	31	0.000
1995	31	0.002
1996	31	0.000
1997	31	0.001
1998	31	0.001
1999	31	0.000
2000	31	0.000
2001	31	0.003
2002	31	0.000
2003	31	0.000
1972	32	0.000
1973	32	0.014
1974	32	0.000
1975	32	0.064
1976	32	0.195
1977	32	0.210
1978	32	1.170
1979	32	4.492
1980	32	17.016
1981	32	9.512
1982	32	11.655
1983	32	14.645

1984	32	12.080
1985	32	10.937
1986	32	4.955
1987	32	3.913
1988	32	1.203
1989	32	0.264
1990	32	0.044
1991	32	0.019
1992	32	0.010
1993	32	0.010
1994	32	0.015
1995	32	0.004
1996	32	0.004
1997	32	0.010
1998	32	0.013
1999	32	0.000
2000	32	0.000
2001	32	0.002
2002	32	0.000
2003	32	0.000
1972	2526	0.000
1973	2526	0.000
1974	2526	0.000
1975	2526	0.000
1976	2526	0.000
1977	2526	0.000
1978	2526	0.000
1979	2526	0.000
1980	2526	0.000
1981	2526	0.000
1982	2526	0.000
1983	2526	0.000
1984	2526	0.000
1985	2526	0.000
1986	2526	0.000
1987	2526	0.000
1988	2526	0.614
1989	2526	0.392
1990	2526	0.833
1991	2526	1.061
1992	2526	0.253
1993	2526	0.061
1994	2526	0.232
1995	2526	1.704
1996	2526	3.081
1997	2526	1.512
1998	2526	1.018
1999	2526	1.453
2000	2526	1.646
2001	2526	1.525
2002	2526	1.526
2003	2526	1.092
2004	2526	0.859
1953	2932	0.086
1954	2932	0.100
1955	2932	0.100
1956	2932	0.100
1957	2932	0.000
1958	2932	0.100
1959	2932	0.000
1960	2932	0.000
1961	2932	0.000
1962	2932	0.027
1963	2932	0.012
1964	2932	0.016
1965	2932	0.023
1966	2932	0.026
1967	2932	0.027
1968	2932	0.070
1969	2932	0.058
1970	2932	0.070

1971	2932	0.053
2005	2932	0.018
2006	2932	0.021
2007	2932	0.017
2008	2932	0.020
2009	2932	0.053
2010	2932	0.051
2011	2932	0.068
2012	2932	0.071
2013	2932	0.070
2014	2932	0.086
2015	2932	0.113
2016	2932	0.148
2017	2932	0.092
2018	2932	0.082
2019	2932	0.066
2020	2932	0.025
2021	2932	0.040
